# Membrane Vesicle Production as a Bacterial Defense Against Stress

**DOI:** 10.3389/fmicb.2020.600221

**Published:** 2020-12-09

**Authors:** Negar Mozaheb, Marie-Paule Mingeot-Leclercq

**Affiliations:** Université catholique de Louvain (UCL), Louvain Drug Research Institute (LDRI), Cellular & Molecular Pharmacology Unit (FACM), Brussels, Belgium

**Keywords:** pathogen, antibiotics, membrane, environmental stress, membrane vesiculation

## Abstract

Membrane vesicles are the nano-sized vesicles originating from membranes. The production of membrane vesicles is a common feature among bacteria. Depending on the bacterial growth phase and environmental conditions, membrane vesicles show diverse characteristics. Various physiological and ecological roles have been attributed to membrane vesicles under both homeostatic and stressful conditions. Pathogens encounter several stressors during colonization in the hostile environment of host tissues. Nutrient deficiency, the presence of antibiotics as well as elements of the host’s immune system are examples of stressors threatening pathogens inside their host. To combat stressors and survive, pathogens have established various defensive mechanisms, one of them is production of membrane vesicles. Pathogens produce membrane vesicles to alleviate the destructive effects of antibiotics or other types of antibacterial treatments. Additionally, membrane vesicles can also provide benefits for the wider bacterial community during infections, through the transfer of resistance or virulence factors. Hence, given that membrane vesicle production may affect the activities of antibacterial agents, their production should be considered when administering antibacterial treatments. Besides, regarding that membrane vesicles play vital roles in bacteria, disrupting their production may suggest an alternative strategy for battling against pathogens. Here, we aim to review the stressors encountered by pathogens and shed light on the roles of membrane vesicles in increasing pathogen adaptabilities in the presence of stress-inducing factors.

## Introduction

Bacterial membrane vesicles are membrane-derived vesicles discharged by both Gram-positive and Gram-negative bacteria. They are heterogeneous in terms of their origin, components, and size. Vesicles derived from Gram-positive bacteria are 20–400 nm in size, while those derived from Gram-negative bacteria are 20–200 nm (Schwechheimer and Kuehn, 2015; Toyofuku et al., 2017). Membrane vesicles are promising vaccine and adjuvant candidates (Ellis and Kuehn, 2010; Jan, 2017). Nonetheless, their production should be strictly controlled when dealing with bacterial infections, primarily because membrane vesicles exert highly deleterious effects on the effectiveness of various antimicrobial agents, intensify the consequences of infections, and are also virulence factors for bacteria (MacDonald and Kuehn, 2012; Chattopadhyay and Jagannadham, 2015; Devos et al., 2017).

Numerous excellent reviews have been published regarding the various functions and characteristics of bacterial membrane vesicles. For instance, Toyofuku et al. (2019) classified membrane vesicles into four distinct types depending on their origin, biogenesis pathway, contents, and functions. Moreover, several studies on factors that trigger the production of membrane vesicles in Gram-negative bacteria have allowed the categorization of these factors, and suggested various vesiculation models in Gram-negative bacteria (Schwechheimer and Kuehn, 2015). Given that membrane vesicle production is conserved among bacteria, and that they have a prominent role in transporting cellular contents, membrane vesicles have been called the type zero secretion system (Guerrero-Mandujano et al., 2017). Membrane vesicles are mediators of bacterial interspecies and cross-species communication, as well as effective mediators of host–microbe interactions, which helps bacteria with colonization and dispersal (Caruana and Walper, 2020). A significant number of studies have also investigated the contribution of membrane vesicles produced by both pathogens and commensals in modulating host immune system responses (Ellis and Kuehn, 2010; Kaparakis-Liaskos and Ferrero, 2015).

### Membrane Vesicles Biogenesis

#### General Mechanisms

**FIGURE 1:**
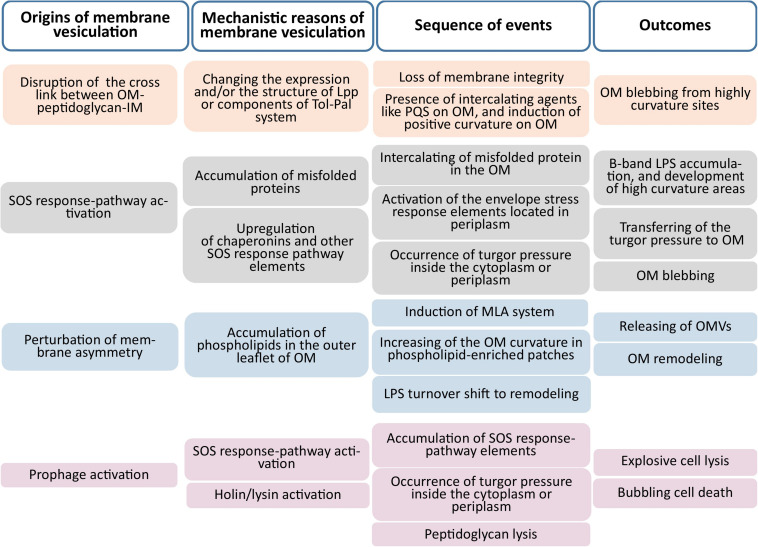
Origins and mechanistic causes of membrane vesiculation. The main four origins illustrated are the disruption of membrane integrity, activation of the SOS-response pathway, perturbation of outer membrane asymmetry, and prophage activation (Outer Membrane; OM, Inner Membrane; IM).

Evidence indicates that the biogenesis of outer membrane vesicles (OMVs) in Gram-negative bacteria relies on four main mechanisms: (i) dissociation of the outer membrane in specific zones lacking proper attachments to underlying structures (e.g., peptidoglycans) (Yeh et al., 2010; Moon et al., 2012); (ii) the presence of misfolded proteins, which accumulate in nano-domains where crosslinks between peptidoglycans and other components of the bacterial envelope are either locally depleted or displaced (Schwechheimer and Kuehn, 2015); (iii) changes in lipopolysaccharide (LPS) structure which presumably results in the generation of a differential curvature, fluidity, and charge in the outer membrane (Elhenawy et al., 2016; Volgers et al., 2018); and (iv) disruption of the maintenance of lipid asymmetry in the outer membrane, based on presence of phospholipids in the outer leaflet of the outer membrane. Figure 1 depicts the origins and mechanistic reasons for membrane vesiculation. The factors underlying the promotion of membrane vesicle production in Gram-positive may be similar to those in Gram-negative bacteria, with membrane vesicles reflecting stress-induced membrane remodeling (Nagakubo et al., 2019). Although a variety of models have been proposed for membrane vesicle production in Gram-negative bacteria, relatively few models have been suggested for Gram-positive bacteria (Brown et al., 2015; Resch et al., 2016).

According to conventional models, membrane vesiculation does not involve cell lysis or death. As this is not supported by all of the experimental evidence (Turnbull et al., 2016; Toyofuku et al., 2017), a new mechanism has been proposed in which vesiculation is the result of explosive cell lysis, or bubbling cell death (Toyofuku, 2019). During outer membrane blebbing, Gram-negative bacterial cells continue their physiologic activities, with vesiculation helping cells to maintain homeostasis. Outer membrane blebbing is accompanied by turnover of the outer membrane, and it resulted in production of OMVs containing LPS, and some vesicles have components of the inner membrane as well. Cytoplasmic turgor pressure can lead to the formation of outer–inner membrane vesicles (OIMVs) (Toyofuku et al., 2019).

Membrane vesicles released after explosive lysis are formed from broken membrane fractions that have reassembled while retaining some cytoplasmic material and periplasm. Induction of endogenous bacterial hydrolases or treatment with an agent that disrupts peptidoglycan synthesis can result in explosive cell lysis (Li et al., 1996; Kawai et al., 2018) and membrane vesicle formation; this has been observed in both Gram-negative and -positive cells (Turnbull et al., 2016; Toyofuku et al., 2017). In the latter, membrane vesiculation may be triggered by an increase in cytoplasmic turgor and/or at a weakened point in the peptidoglycan layer of the cell wall, through which the cytoplasmic membrane and part of cytoplasm pass to form membrane vesicles (Brown et al., 2015). Such vesiculation often leads to cell death and is referred to as bubbling cell death (Toyofuku et al., 2019). Although there are mechanistic differences between bubbling cell death and vesiculation in Gram-positive bacteria caused by explosive cell lysis, in practice there is no clear distinction between vesicles produced through the two processes.

#### Role of Inner Membrane for OMV Production

Studies based on stochastic as well as non-stochastic models of membrane vesiculation have shown that it is a highly regulated process. Most studies to date on membrane vesiculation have focused on Gram-negative bacteria (and hence, OMVs). However, recently there has been increasing interest in the regulatory role of the inner membrane (Davies et al., 2019; Takaki et al., 2020). The role of inner membrane in outer membrane vesiculation could be considered via various aspects, including (I) Inner membrane regulates and keeps the cross-talk between the components of the bacterial envelope, e.g., outer membrane-peptidoglycan-inner membrane, (II) Inner membrane regulates the stress response-pathway, (III) Inner membrane contains maintenance elements for outer membrane asymmetry (IV) Inner membrane can convey the turgor pressure of misfolded proteins and SOS response-pathway to periplasm and outer membrane. The following paragraphs present the explanation of each role.

(I) Inner membrane and envelope integrity. Correct localization of lipoproteins is critical for their function and depends on efficient modification and transport as well as accurate sorting of lipoproteins. All lipoproteins are translated in the cytoplasm as preprolipoprotein precursor proteins. After their export through the cytoplasmic membrane, which occurs predominantly but not exclusively via the general secretory (Sec) pathway, the proteins are modified with lipids at the cytoplasmic membrane in a multistep process (Zückert, 2014). Depending on *N*-acylation and specific sorting signals, Lpp (the best known crosslinked lipoprotein) either remains in the inner membrane or is translocated to the inner leaflet of the outer membrane via the Lpp outer membrane localization pathway (Kaplan et al., 2018), after which it remains at the periplasmic site or is transported to the cell surface (Knoke et al., 2020). Stress can alter the structure of peptidoglycan or Lpp, leading to membrane blebbing due to the loss of membrane integrity; the balance between peptidoglycan turnover and elements that preserve membrane integrity through lipoprotein crosslinking determines the degree of membrane vesiculation (Martins et al., 2019; Ultee et al., 2019). Moreover, the presence of diacylated Lpp induces specific attachment and internalization of OMVs by target bacteria (Knoke et al., 2020).

There is also another mechanism for keeping envelope integrity named Tol-Pal system. The Tol-Pal system is involved in cell division and maintenance of cell wall integrity in Gram-negative bacteria. The system in *E. coli* contains 5 elements. Of these, TolA, TolQ, and TolR are transmembrane proteins located in the inner membrane; the periplasmic domain of TolA interacts with the periplasmic protein TolB, which directly interacts with Pal, a lipoprotein anchored in and connecting the outer membrane to peptidoglycans through non-covalent interaction. This system links the outer and inner membranes, and loss of function of its components compromises membrane integrity (Gerding et al., 2007), leading to hyper vesiculation (Takaki et al., 2020). Disruption of the cell envelope and detachment of the outer from the inner membrane is a major factor contributing to membrane vesiculation (Schwechheimer et al., 2013). Membrane vesicles released in this manner are likely to be OIMVs (Takaki et al., 2020).

(II) Inner membrane and stress response pathway. The inner membrane plays a critical role in the stress response. For example, the conjugative plasmid expression (CPx) response (McEwen and Silverman, 1980) is induced by a variety of signals including inner membrane protein folding stress and NlpE-dependent signals, resulting in the autophosphorylation of CpxA, which then phosphorylates and activates the response regulator CpxR for transcriptional regulation (Mitchell and Silhavy, 2019). This system is analogous to the envelope stress sigma factor (σ^E^) response to outer membrane stress in *E. coli* (Alba and Gross, 2004). *P. aeruginosa* AlgU is a homolog of the *E. coli* heat shock sigma factor RpoE that positively regulates the synthesis of B-band LPS, which reduces cell surface hydrophobicity and inhibits outer membrane blebbing at sites of B-band accumulation (Murphy et al., 2014). Defects in protein secretion across the inner membrane are thought to serve as a signal for Cpx activation (Wall et al., 2018), although the relationship between Cpx-activating stress and protein misfolding has yet to be elucidated (Mitchell and Silhavy, 2019).

(III) Inner membrane and envelope asymmetry. The inner membrane is a key element in the maintenance of the membrane lipid asymmetry (MLA) pathway regulating membrane vesiculation (Davies et al., 2019). In the asymmetric outer membrane, the outer leaflet harbors lipopolysaccharides whereas the inner leaflet is mostly composed of phospholipids. The presence of phospholipids in the outer leaflet of the outer membrane can activate the MLA pathway, which includes an inner membrane ATP-binding cassette (ABC) transporter consisting of MlaFEDB, the periplasmic chaperone MlaC, and the outer membrane lipoprotein MlaA. Stressors such as starvation or high salt concentration can alter the expression of MLA system components, leading to phospholipid accumulation in the outer membrane. Additionally, an increased abundance of phospholipids in the outer leaflet of the outer membrane induces LPS remodeling, which is facilitated by membrane vesiculation through acceleration of membrane turnover and leads to budding from areas of the outer membrane with high phospholipid concentration (Roier et al., 2016). The presence of nutrient-absorbing molecules on the surface of OMVs induced by starvation enhances the dispersal of these molecules in the environment. Upon nutrient deficiency, the cell downregulates components of the MLA system (Manning and Kuehn, 2011; Zingl et al., 2020), resulting in the release of membrane vesicles with nutrient-absorbing molecules such as iron chelators on their surface (Roier et al., 2016; Davies et al., 2019).

(IV) Inner membrane as a mediator of turgor pressure. In Gram-negative, the inner membrane functions as the first sensor of turgor pressure and intercalator of molecules in the outer membrane. Stress increases the concentration of unfolded proteins in the cytoplasm and periplasmic area. Turgor pressure within the cytoplasm can lead to outer membrane blebbing and budding or explosive cell lysis. Vesicles formed after stress induction likely contain stress response factors. The activity of chaperonins such as proteases reduces cytoplasmic pressure by degrading unfolded proteins, thus controlling membrane vesiculation (McBroom et al., 2006; Toyofuku et al., 2019). The *Pseudomonas* quinolone signal (PQS) is an example of a molecule that intercalates into the outer membrane. Interestingly, PQS appears to be located in the inner membrane in the low–OMV-producing *P. aeruginosa* strain PAO1, unlike in strains producing larger numbers of OMVs. Under conditions of stress, *P. aeruginosa* activates the SOS response with upregulation of the PQS, which localizes in the outer membrane where it sequesters Mg^2+^ and Ca^2+^ and destabilizes LPS. Anionic repulsion between LPS and PQS induces curvature of the outer membrane at sites of PQS accumulation, resulting in outer membrane blebbing (MacDonald and Kuehn, 2013; Jan, 2017). PQS accumulation in the inner membrane is a hallmark of poor OMV producers (Florez et al., 2017).

### Functions of Membrane Vesicles

**FIGURE 2:**
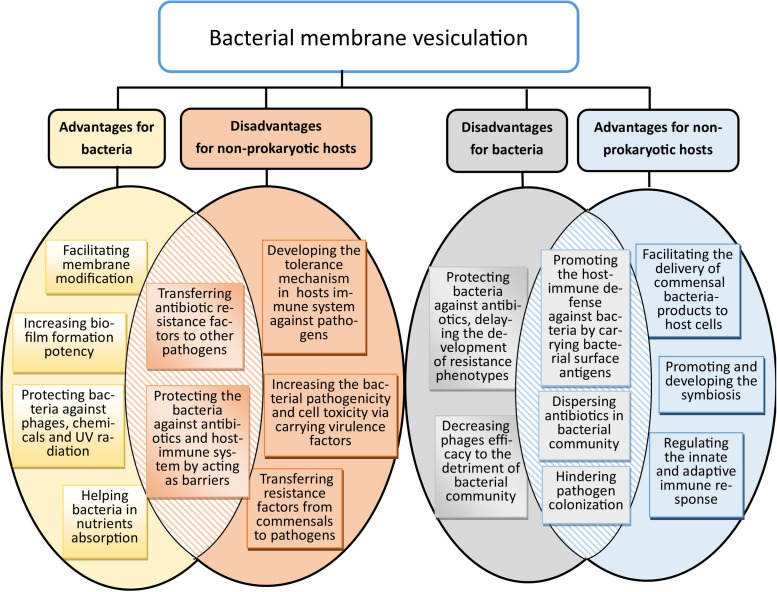
Benefits and drawbacks of bacterial membrane vesiculation for bacteria and non-prokaryotic hosts. Some of their advantages for bacteria could directly hit harm to non-prokaryotic hosts, and vice versa.

Multiple functions have been attributed to membrane vesicles in both interbacterial interactions and host–bacterial. They include (i) the activation of the host immune response through pattern recognition receptors; (ii) establishment of ecological niches by facilitating predatory activity through the delivery of active enzymes to adjacent bacteria (Li et al., 1998); (iii) facilitation of interbacterial communication, for instance via the transfer of quorum-sensing auto-inducers as well as horizontal gene transfer (Toyofuku et al., 2017); (iv) defense against environmental insults by acting as decoys that prevent antibiotics, antimicrobial peptides, and bacteriophages from reaching the bacterial cell (Manning and Kuehn, 2011); and (v) enabling biofilm formation, thereby rendering bacteria more tolerant to environmental stressors (Kim and Davey, 2020). From various aspects, bacterial membrane vesiculation is in favor of bacteria and their non-prokaryotic hosts, while it could cause serious detrimental effects on both (Figure 2).

Bacteria have evolved a diverse collection of adaptive mechanisms that enable them to survive and grow in the presence of different environmental stresses. The production of membrane vesicles is one approach for attaining an adapted phenotype, or may be the result of other stress responses that lead to membrane vesiculation. Some bacterial responses to stressors are long-term adaptations, and are dependent on transcriptional regulation, induction of anabolic pathways, and cell growth. Others are short-term responses, such as those required for surviving sudden environmental changes, including heat shock or the presence of toxic organic solvents (Eberlein et al., 2019). Bacteria respond to stress via one or a combination of these responses, depending on the type of stress. For instance, in human tissue, pathogenic bacteria are exposed to various stresses such as unfavorable pH, osmotic pressure, temperature extremes, limited nutrient availability, and the presence of antibiotics (Ultee et al., 2019). To survive in these aggressive conditions, pathogens need to fine-tune their phenotypes (Martins et al., 2019). Living in a microbial community may also represent a stressful condition for bacteria due to competition within multi-microbial as well as single-species communities, and between host microbiota and pathogens (Cecil et al., 2019). Under these conditions, bacteria try to become the dominant community of their ecological niche through the production of bacteriocins, endolysins, quorum-sensing signal molecules, and bacterial toxins and enzymes that target other bacterial species and eukaryotic cells and tissues (Caruana and Walper, 2020). Moreover, they can change their physiology to develop their niches, such as happens with *Pseudomonas aeruginosa* that establishes an anaerobic population in the lower layer of a stratified multi-microbial biofilm with *Staphylococcus aureus* (del Mar Cendra et al., 2019).

Antibiotics are prominent stressors and can be considered as the most potent inducers of defense mechanisms in pathogens (Ultee et al., 2019). Thus, bacteria develop various and specific resistance strategies such as establishing an impermeable envelope (Simpson and Trent, 2019), changing the molecular structure of antibiotic targets (Emami et al., 2017), modifying the chemical structure of antibiotics (Wright, 2005), and pumping antibiotics out of the cell (Alcalde-Rico et al., 2016), while at the level of microbial communities, they transfer resistance genes and enzymes to the surrounding environment as well as each other (MacDonald and Kuehn, 2012; Schwechheimer and Kuehn, 2015). Moreover, following exposure to antibiotics, bacteria tend to develop a structured community, such as a biofilm (Alam et al., 2019).

Because membrane vesicle overproduction has been detected in most cases of stress-activated defense mechanisms, they are thought to be a form of bacterial defense against stress. Membrane vesicles can help the bacteria cope with multi-stress conditions via either proven or speculated mechanisms (McBroom et al., 2006; McBroom and Kuehn, 2007; MacDonald and Kuehn, 2012). Nonetheless, in many instances, the contribution of membrane vesicle generation, as well as its consequences, remains elusive and enigmatic. Moreover, whether their production is completely regulated, or is partially stochastic, also remains undetermined (Orench-Rivera and Kuehn, 2016).

**FIGURE 3:**
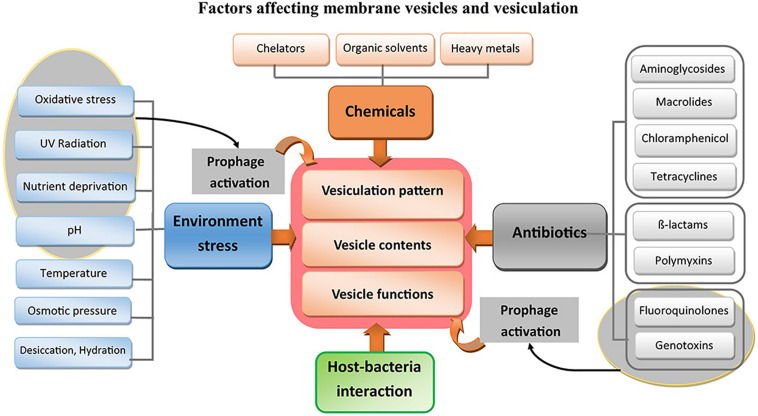
Inducers of vesiculation in bacteria. The stresses that change the vesiculation in bacteria categorize into five major groups; (I) Environmental abiotic stresses, (II) Groups of antibiotics, (III) Chemical treatments, (IV) Prophage effects, and (V) effects of interactions between bacteria and their host.

In this review, we discuss the stressors that induce changes in vesiculation patterns and how bacteria benefit from these changes to survive environmental insults. The studies that have been performed on the environmental stresses, antibiotics, chemical threats, and phage and prophage activities that affect membrane vesiculation are summarized in Supplementary Table 1. The bacterial stressors that can alter membrane vesiculation patterns, membrane vesicle contents, or membrane vesicle functions are illustrated in Figure 3. Each section of this review is dedicated to one type of stress and the probable changes in vesiculation patterns that occur in studied bacteria under that stress. Although pathogens may not be directly exposed to some of the mentioned insults, highlighting how bacteria exploit membrane vesicles to defend against environmental stresses could help establish an extensive perspective on the physiological and the protective functions of membrane vesicles in pathogens. Hence, this review gives an insight into the studies undertaken on membrane vesicles and allows for more comprehensive speculation on ways to control vesiculation to constrain infectious diseases and antibiotic resistance.

## Environmental Stress

Environmental stress refers to abiotic factors that promote unfavorable conditions for bacteria, leading to a decrease in optimal growth and viability. The production of membrane vesicles is highly influenced by environmental stresses. Most stressors that promote membrane vesicle production benefit bacterial cells either directly, such as by exporting misfolded proteins to the outside of the cell; or indirectly, such as in outer membrane blebbing following changes in outer membrane composition. Under environmental stresses, bacteria can change their vesiculation pattern as an immediate protective response (Manning and Kuehn, 2011).

### Oxidative Stress

Bacteria are affected by oxidative stress in their ecological niches. Several factors can cause oxidative stress, including interaction with the immunity system of their hosts (e.g., macrophage oxygen burst), the availability of free radicals in their microenvironments, the presence of ferric ion (Fe^3+^), a deficiency of electron donors such as glutathione, and treatment with antimicrobial agents. Under oxidative stress, bacterial stress response elements such as heat shock proteins, starvation response elements, and SOS pathway elements are extensively induced, and membrane vesiculation is triggered (Storz et al., 1990; Farr and Kogoma, 1991; Gerritzen et al., 2018). In this regard, various examples have been reported. HtrA and DsbI are serine protease (Heimesaat et al., 2014) and thioredoxin (Landeta et al., 2018), respectively, and both have chaperonin like activity hence, stress induces their production. In *Campylobacter jejuni*, oxidative stress leads to an overall increase in OMV production, while, mutants in chaperonin-coding genes (i.e., Δ*htrA* and Δ*dsbl*) do not show an increase in OMV production under stress conditions (Godlewska et al., 2019). Toyofuku et al. (2014) showed that OMV production is increased in *P. aeruginosa* PAO1 under anoxic conditions that lead to the production of intermediate radicals of denitrification respiration. PrtN, the major activator of the SOS pathway in *P. aeruginosa*, is induced by nitrogen free radicals and promotes the activation of this pathway (Matsui et al., 1993). Additionally, SOS pathway activation can induce the production of F- and R-pyocins, and the whole or a part of these prophages are observed as components of OMVs (Toyofuku et al., 2014). Moreover, in *E. coli*, the OxyR protein has been shown to act as a regulatory element for some oxidative stress response genes (Gundlach and Winter, 2014), and its deletion causes a reduction in OMV production in wild-type *E. coli* (Kulp et al., 2015). In *Neisseria meningitides*, the OMV transcriptomic data obtained under cysteine-depleted conditions were similar to those of OMVs released *in vivo*, as well as under oxidative stress (van de Waterbeemd et al., 2013). In *N. meningitidis*, applying external stress like a high amount of dissolved oxygen, up to 150%, decreased the growth rate of bacteria, while effectively increasing OMV production (Gerritzen et al., 2018).

Although numerous examples have illustrated the relationship between the induction of oxidative stress responses and hypervesiculation, the response may depend on the nature of the oxidative stress. Cysteine and iron maintain redox homeostasis through regulation of the expression of oxidoreductase enzymes, such as superoxide dismutase (Deng et al., 2013). Therefore, a shortage of cysteine and iron can mimic conditions of oxidative stress, restricting the growth of microorganisms, and can also induce the expression of some elements of the SOS pathway (Turnbull and Surette, 2010; Deng et al., 2013; van de Waterbeemd et al., 2013; Chan et al., 2017). High concentrations of free metal ions in the environment can threaten bacterial survival. Interestingly, to limit their toxicity, in addition to activating stress response pathways, bacteria can also immobilize chemical elements on their cell surface, which leads to membrane blebbing. This would explain why passive nucleation and metal precipitation have been associated with the generation and release of OMVs from the surface of *Leptospira interrogans*, as well as its stress response metalloprotease *Htpx* knockout mutant (Henry et al., 2013).

Outer membrane modifications constitute a defensive mechanism against oxidative stress in bacteria, and can result in membrane vesicle production (McBroom and Kuehn, 2007). Indeed, OMV production is tightly dependent on the O-antigen composition under both homeostatic and stress conditions (MacDonald and Kuehn, 2013). O-antigen is one of the most important virulence factors for Gram-negative bacteria, and the pathogenicity of this factor is due to its interaction with host immune systems (McGroatry and Rivera, 1990; Moran, 2001; DebRoy et al., 2011; MacDonald and Kuehn, 2013). Murphy et al. (2014) targeted three main genes in the LPS biosynthesis pathway to reveal the effect of O-antigen composition on membrane vesiculation in *P. aeruginosa*. They observed that Δ*wbpL* strains, which harbored a deletion of the initial glycosyltransferase for synthesizing A-band and B-band LPS, produce larger OMVs. Meanwhile, Δ*rmd* and Δ*wbpM* strains that are defective for the biosynthesis of A- and B-band LPS, respectively, produce smaller OMVs (Murphy et al., 2014). A-band regions are in low curvature areas of the outer membrane, whereas B-bands exist mostly in areas of high curvature. The accumulation of an electronegative charge in B-band areas creates an electrostatic repulsion that increases the curvature of these parts. Finally, these high curvature areas bleb, and OMVs are released (Kadurugamuwa and Beveridge, 1996). MacDonald and Kuehn showed that oxidative stress-induced OMV production in *P. aeruginosa* requires the presence of enough B-band-enriched regions in the bacterial outer membrane (Sabra et al., 2003; MacDonald and Kuehn, 2013). The role of outer membrane modifications in defending against oxidative stress has also been investigated by Sinha et al. (2019). They observed that in *Citrobacter rodentium*, as in other enteric bacteria, a two-component system (PmrAB/C) as well as the CptA protein maintain membrane integrity against various stresses. These proteins become upregulated in the presence of high iron concentrations, and they modify the LPS to reduce the adverse effect of a high iron concentrations. Activation of this system negatively regulates OMV production; hence, in Δ*pmrAB*, Δ*pmrC*, and Δ*cptA* mutants, a high iron concentration induces OMV formation (Sinha et al., 2019). Furthermore, under oxidative stress, the concentration of misfolded proteins in the periplasmic environment increases, and bacteria expel these misfolded proteins by packing them into membrane vesicles in balance with chaperonin activity; membrane vesicle production levels off when the chaperonins become highly expressed (MacDonald and Kuehn, 2013).

### UV Radiation

UV radiation exerts its toxic effect by triggering the formation of free radicals, as well as inducing prophage activities (Kageyama et al., 1979; Courcelle et al., 2001; Toyofuku et al., 2014). Prophage activation is an effective mechanism for membrane vesicle formation in both Gram-positive and Gram-negative bacteria (Toyofuku et al., 2014). In *Bacillus subtilis*, membrane vesicle production is dependent on prophage-encoded endolysin (Toyofuku et al., 2017). Endolysin is a peptidoglycan hydrolase that is transported to the periplasm by holin, an integral membrane protein (Nakayama et al., 2000). *Bacillus subtilis* 168 contains two major prophage systems, PBSX and the SPβ holin–endolysin system (Kunst et al., 1997). In this microorganism, holin activity is activated in the presence of genotoxic insults (e.g., UV radiation) and directs endolysin to degrade the peptidoglycan; subsequently, the cytoplasmic membrane is forced out through the created hole, and a membrane vesicle is released (Toyofuku et al., 2017). UV-induced membrane vesicle production has also been observed in freshwater bacteria, and is considered a protective response against high UV radiation (Gamalier et al., 2017). In a study on non-pathogenic bacteria, Zarantonello et al. (2018) investigated the effect of a detrimental dose of UV radiation on a cyanobacterium, *Cylindrospermopsis raciborskii*, and observed that UV radiation could induce membrane vesicle genesis.

### Nutrient Deprivation

Starvation can induce extensive stress response pathways in bacterial cells (Wonderling et al., 2004). Iron limitation, cysteine deficiency, and magnesium depletion are instances of nutrient depletion that promote the general stress response pathways. Inside the hosts, nutrient depletion can also induce pathogen colonization, as well as the expression of virulence factors, followed by the triggering of membrane vesiculation (Bishop and Work, 1965; van de Waterbeemd et al., 2013; Zingl et al., 2020).

The limitation of free iron during colonization inside their hosts is growth-limiting for pathogens. This limitation can increase the concentrations of general stress response proteins, such as UvrA and UpsF, in membrane vesicles secreted by enterotoxigenic *Escherichia coli* (ETEC) (Chan et al., 2017). Some bacteria can benefit from specific components inside membrane vesicles. For example, OMVs produced by *Porphyromonas gingivalis* contain the HmuY protein, which possesses heme-binding activity, allowing the bacterium to absorb enough iron in nutrient-deprived environments, such as in biofilms. Not only do bacteria put this protein on the surface of their outer membrane, but they can also release it in soluble form to the medium of their ecological niche (Olczak et al., 2010; Bonnington and Kuehn, 2014). Lin et al. (2018) proposed a model for iron uptake in *P. aeruginosa* that is dependent on the presence of PQS molecules on the surface of the OMVs, as well as a ligand for PQS (TseF) secreted by the type VI secretion system. According to this model, TseF interacts with PQS-associated iron on the OMVs on the one hand, while on the other, it can be transferred to other bacteria that express FptA, a receptor for this ligand. Although this model was proposed for *P. aeruginosa*, it is thought that various other bacteria exploit OMVs for iron uptake in a similar manner (Lin et al., 2017). Iron acquisition via exploiting membrane vesicles also has been observed in Gram-positive bacteria. *Mycobacterium tuberculosis* faces serious iron deficiency in their hosts. To cope with this stress, they produce siderophores and release them to their environment packed in membrane vesicles. The overproduction of membrane vesicles carrying mycobactin was observed in *M. tuberculosis* cultured in minimal media with a limited iron concentration (Prados-Rosales et al., 2014). The system for maintaining outer membrane asymmetry has two major components; VacJ/Yrb ABC, an ATP-binding cassette, and phospholipid transferase. Former transfer phospholipids from the outer leaflet of the outer membrane to the inner leaflet, and later transfer phospholipids to the outer leaflet of the outer membrane. This system contributes greatly to cell responses to starvation by controlling membrane vesiculation, and Ferric Uptake Regulator (FUR) is one of the regulators of this system. Roier et al. revealed that, in the presence of iron, FUR positively controls the expression of the VacJ/Yrb ABC transporter and downregulates that of phospholipid transporters (Roier et al., 2016). Thus, in the absence of iron, hypervesiculation occurs because membrane asymmetry is disrupted (Malinverni and Silhavy, 2009; Hassan and Troxell, 2013). Moreover, at the initial stages of infection when pathogens are faced with iron deficiency in their hosts, they switch off this system so as to better adapt to their host environment via hypervesiculation as well as LPS modification (Zingl et al., 2020).

Similar to cysteine depletion (van de Waterbeemd et al., 2013), sulfur shortage also induces oxidative stress, following which the membrane asymmetry maintenance system (VacJ/Yrb ABC transporter) is downregulated. Moreover, sulfur depletion results in the overproduction of NADPH, which is required for the production of phospholipids from serine. Together, these events perturb outer membrane asymmetry, causing outer membrane protrusion and, consequently, OMV formation (Gerritzen et al., 2019).

In *Salmonella enterica*, the PhoPQ system is induced through Mg^2+^ deficiency. PhoPQ positively regulates the levels of the PagL protein, which is responsible for lipid A deacetylation. This modification promotes a higher curvature in the outer membrane and triggers membrane blebbing (Elhenawy et al., 2016; Sinha et al., 2019). Furthermore, in OMVs resulting from SOS pathway activation in *P. aeruginosa*, the outer membrane protein OprH was found to be regulated by Mg^2+^ starvation, indicating that Mg^2+^ shortage, as well as SOS pathway activation, can both promote OMV production (Maredia et al., 2012).

### pH

Both the production rate and content of bacterial membrane vesicles can be altered by pH changes. Several fold increases in vesiculation rates have been observed at low pH for both extracellular and facultative intracellular bacteria, including *F. tularensis* (Klimentova et al., 2019), *Salmonella typhimurium* (Malabirade et al., 2018), and *S. enteridis* (Chart et al., 1994).

Under unfavorable changes in pH, membrane vesicle generation is beneficial for the biophysical modifications required for the maintenance of membrane integrity. In *S. enterica*, shifting from pH 7.6 to 5.8 results modifications in lipid, including the addition of 4-amino-4-deoxy-L-arabinose (L-Ara4N) and zwitterionic phosphatidylethanolamine (pEtN) to the phosphates of lipid A, and palmitoylation of the acyl chain. These changes increase the outer membrane stabilization and help the bacteria to alleviate membrane damage. Interestingly, OMVs were depleted of lipid A with modified phosphate moieties likely enabling outer membrane remodeling toward an environmentally adapted LPS composition. Deviations in the observed OMV lipid A content from the stochastic OMV formation model under mildly acidic environmental conditions with unexpected retention of more highly modified lipid A species in the outer membrane might result from the less energy required to synthesize unmodified lipid A (Chart et al., 1994; Bonnington and Kuehn, 2016). Additionally, acidic pH and the resulting modifications in outer membrane and LPS composition can affect the activity of proteins that modulate the levels of covalent cross-linking of peptidoglycans to the outer membrane via the lipoprotein Lpp (Schwechheimer et al., 2015). Additionally, in acidic pH, changes in the expression of outer membrane proteins (OMPs) and flagella have been observed in *Salmonella enteritidis* (Chart et al., 1994).

How modulation of pH of colonized tissues affects the interactions between bacteria and host cells, and the mechanisms through which hosts modulate the chemistry of symbiosis to regulate microbial community function, are poorly understood. For instance, the marine bacterium *Vibrio fischeri*, which forms a specific mutualism with the Hawaiian bobtail squid, *Euprymna scolopes*, upregulates the transcription of its major OMP, OmpU, during growth at an acidic pH (Lynch et al., 2019). OmpU is a size-selective porin that dynamically dilates and constricts, likely to deliver specific cargo molecules to host tissues. ToxR, H-NS, and OmpR are regulators of OmpU expression. Under the acidic pH of the host, *V. fischeri*-derived OMVs become more potent stimulators of symbiotic host development in an OmpU-dependent manner, promoting light organ development in the squid. The development of this symbiosis can be stimulated by OMVs containing a homolog of OmpU from the pathogenic species *Vibrio cholerae* (Lynch et al., 2019).

The effects of pH in cell–cell communication are also dependent on the composition of OMVs, as demonstrated for *S. enterica* with the different effects being dependent on the release of less modified and more highly acylated lipid A structures. This could act to manipulate the host immune system response, whereby the structure most encountered by immune cells would not be representative of the actual membrane composition of the invading cell (Bonnington and Kuehn, 2016).

### Temperature

Bacteria are affected by heat-shock stress. Katsui et al. (1982) successfully increased membrane blebbing events in *E. coli* W3110 by heating the bacteria at 55°C for 10 min. While evaluating the role of membrane vesicles in the increase of pathogenicity, Baumgarten et al. (2012a) studied the effect of heat shock on OMV contents and the rate of OMV production in *Pseudomonas putida* DOT-T1E. They observed that high-temperature stress could lead to overproduction of OMVs and increased membrane hydrophobicity, which increased the tendency for biofilm initiation by the microorganism (Pasmore et al., 2001; Baumgarten et al., 2012a).

High-temperature stress can induce changes in the composition of the outer membrane. When *P. aeruginosa* PAO1 are grown at 45°C, they tend to not produce O-antigen (R-formed LPS) (Kropinski et al., 1987; Makin and Beveridge, 1996). Additionally, mutant *P. aeruginosa* PAO1 strains that cannot produce O-antigen release significantly larger OMVs (Murphy et al., 2014). Envelope stress response elements, such as σ^E^ in *E. coli* (Alba and Gross, 2004) or AlgU in *P. aeruginosa* (Tashiro et al., 2009), as well as proteasome systems such as HtrA in *C. jejuni* (Boehm et al., 2018) or DegP in *E. coli* (Walsh et al., 2003), have been extensively studied in relation to the effects of heat-shock stress. AlgU is also highly effective at promoting the initiation of OMV production (Tashiro et al., 2009). Hence, activation of envelope stress response pathways seems to be needed for stress-induced membrane vesicle production in bacteria (MacDonald and Kuehn, 2013; Godlewska et al., 2019). High-temperature stress also leads to the production of OMVs carrying enzymes necessary for the biosynthesis and metabolism of bacterial envelope components, which may reflect the role of envelope modifications in membrane vesicle production in bacteria (Klimentova et al., 2019).

High temperature also seems to affect the percentage of fatty acid saturation in the bacterial cell, and promotes the production of saturated fatty acids as well as OMVs with a higher concentration of saturated fatty acids (Kropinski et al., 1987; Baumgarten et al., 2012a). Additionally, the conversion of *cis*-unsaturated fatty acids into their corresponding *trans* configurations represents a strategy to withstand elevated temperatures in *Vibrio* sp. strain ABE-1 (Diefenbach et al., 1992). An increased *trans/cis* ratio results in a more rigid membrane (Diefenbach et al., 1992; Chen et al., 1995). Indeed, under non-stressed conditions, unsaturated fatty acids exhibit a *cis* configuration, and the double bond present displays an unmovable bend of 30° (Tyler et al., 2019). This kink leads to steric hindrance within the fatty acid residues, thereby enhancing membrane fluidity. In contrast, in *trans* unsaturated fatty acids, the kink is only 6° (Macdonald et al., 1985). Thus, compared with their *cis* isomers, *trans* fatty acids can align more closely with each other and, in this regard, resemble saturated fatty acid residues (Heipieper et al., 2003; Pierce et al., 2014; Kulig et al., 2016).

In *P. aeruginosa*, heat shock stress can also lead to an increase in the production of PQS, in addition to that of OMVs (Mashburn-Warren et al., 2008). Given the highly hydrophobic structure of PQS and its role as an iron chelator, PQS can replace the Mg^2+^ and Ca^2+^ of the outer leaflet of the outer membrane with iron, and change membrane asymmetry (Bredenbruch et al., 2006; Mashburn-Warren et al., 2008; Roier et al., 2016). Consequently, PQS can induce membrane curvature and cause membrane blebbing (Mashburn-Warren et al., 2008). Tashiro et al. (2009) reported that, in *P. aeruginosa*, heat shock stress can induce changes in the conformation of OMPs, thereby activating the expression of periplasmic proteases such as MucD. Tashiro et al. (2009) reported that, in *P. aeruginosa*, heat shock stress can induce changes in the conformation of OMPs, thereby activating the expression of periplasmic proteases such as MucD. Moreover, diminished levels of misfolded proteins via MucD can negatively regulate the production of OMVs, and a high level of MucD decreases the presence of PQS packed in OMVs but, the deletion of *mucD* does not change the expression of PQS. Hence, MucD regulates the PQS placement inside the OMVs (Tashiro et al., 2009). However, neither the level of PQS production nor activation of the envelope stress response system under acute stress is an indicator of the level of OMV production (MacDonald and Kuehn, 2013).

Although pathogenic bacteria frequently encounter high-temperature stress, the opposite is also true, and several studies have investigated the effect of low temperature on membrane vesicle production. Klimentova et al. compared the effects of high temperature and low temperature on OMV production in *Francisella tularensis*, and observed that high temperature is stronger inducer for membrane vesiculation than low temperate stress (Klimentova et al., 2019). Several studies have also shown that prompt membrane vesiculation in low temperature can help bacteria adapt to their ecosystems (Nevot et al., 2006; Frias et al., 2010).

### Osmotic Pressure, Desiccation, and Hydration

Hyperosmotic pressure and desiccation are two distinct environmental stresses. The former refers to a condition in which the medium surrounding the bacteria has lower water activity (a_w_) than that of bacterial environments, while the latter is a condition where the surfaces of their cell walls are exposed to a gas phase. Both types of stress can affect bacterial viability by disturbing their homeostatic condition, leading to adverse effects such as reduced membrane fluidity, changes in protein folding, and changes in genomic structure (Burgess et al., 2016; Esbelin et al., 2018). Hence, bacteria respond to hyperosmotic pressure and desiccation by activating the general stress response pathway, which importantly includes the protease system. Similar to that seen under heat shock stress, Baumgarten et al. (2012a) observed that OMV release increases in *P. putida* under osmotic pressure. The strains in which membrane vesiculation is triggered by osmotic pressure gain a higher biofilm-producing capacity (Baumgarten et al., 2012a). In *Listeria monocytogenes*, HtrA activation decreases the concentration of misfolded proteins that occurs in response to a high salt concentration in the environment (Wonderling et al., 2004). In *P. aeruginosa*, the expression of the osmotically inducible lipoproteins OsmC and OsmE is under the control of the major mediator of the envelope stress response, AlgU (Wood et al., 2006). Hence, the presence of osmotic pressure followed by activation of periplasmic proteases can regulate membrane vesicle production (Tashiro et al., 2009). In contrast, McBroom et al. revealed that, in *E. coli*, osmotic pressure does not directly control membrane vesicle production, given that Δ*ompR* mutant strains, which are defective for the osmotic response regulator protein OmpR, did not show significant changes in OMV production (McBroom et al., 2006).

Under osmotic stress, bacteria develop several direct or indirect lines of defense including changes in lipid membrane composition. As such, the acyl chain composition of lipid A from LPS, phosphatidylethanolamine (PE), phosphatidylglycerol (PG), and cardiolipin (CL) is modified with, for instance, fatty acids with odd numbers of carbon atoms (likely cyclopropane fatty acids), which promotes a shift from a lamellar to a non-lamellar inverted state (Sutton et al., 1991; Brandenburg et al., 1992, 1993). Interestingly, inverted hexagonal structures promote membrane fusion (Siegel, 1999), likely to facilitate membrane vesicle genesis. Non-lamellar structures also are required for efficient folding of the outer membrane porin PhoE of *E. coli* (de Cock et al., 1999). Additionally, changes in fatty acid chains might modify interdigitation and, in turn, membrane order and membrane vesicle genesis (Danner et al., 2008). To maintain membrane fluidity, bacteria benefit from membrane phospholipid phase modifications. Hence, under conditions of dehydration or osmotic pressure, the membrane transits from the liquid-crystalline phase to the gel phase via modifications in phospholipid composition (Mille et al., 2002). If changes in membrane lipids can directly influence membrane properties, including lipid phases, membrane fluidity, membrane tension, and interfacial curvature, indirect effects can result from the annular lipids that directly contact an integral membrane protein. Changes in lipid structure or the physical properties of the membrane can influence the function of membrane osmoregulatory proteins; for instance, in *E. coli*, when osmotic stress is imposed, the proportion of CL increases as the proportion of PE decreases (Romantsov et al., 2009). Osmotic induction of the gene encoding CL synthase (*cls*) contributes to these changes by modulating the activity of osmoregulatory proteins such as MscL, ProP (Romantsov et al., 2009), and AqpZ (Laganowsky et al., 2014).

Furthermore, cells maintain their normal membrane lipid structure under both osmotic and desiccation stresses with the aid of compatible solutes that can fill the spaces around cells, hindering cell collapse and forming hydrogen bonds with the head groups of membrane phospholipids. Membrane vesicles can transfer the compatible solutes to the extracellular environment. The accumulation of compatible solutes in the periplasm can result in outer membrane turgor pressure and vesiculation in Gram-negative bacteria (Gerhardt et al., 1996). When exposed to a gas that has low a_w_ compared with the cell compartments, bacteria efflux water to the extracellular environment; additionally, under osmotic pressure, cells lose water to reach an osmotic equilibrium with the environment. Hence, both osmotic and desiccation stresses cause a decrease in cell volume, and a perturbed area-to-volume ratio of the membrane can result in membrane vesiculation (Mille et al., 2002).

Notably, when the volume of spherical vesicles is experimentally reduced, mimicking osmotic deflation, the vesicles undergo greatly differing shape transformations. This polymorphism is due to the redistribution of a small fraction of the lipids between the inner and outer leaflets of the bilayered membranes thereby, reduce the overall bilayer tension and, produce tensionless bilayers (Ghosh et al., 2019).

In addition to osmotic pressure and desiccation stress, hydration stress can also induce cell membrane vesiculation; moreover, the kinetics of hydration is a determinant factor in cell vesiculation and the intensity of the hydration-induced stress. Thus, the higher the rate of hydration, the greater the level of vesiculation. As previously mentioned, the phospholipid phase transition is an adaptive strategy under desiccation and osmotic pressure stresses; indeed, if rehydration occurs rapidly, compatible solutes leak out of the cell, and viability is decreased (Crowe et al., 1988; Mille et al., 2002).

## Antibiotics

Given the defensive, ecological, and physiological roles of membrane vesicles, such as horizontal interspecies gene transfer and increasing membrane hydrophobicity, antibiotics with inductive effects on membrane vesicle production might not act efficiently in curing bacterial infections since induced membrane vesicle production increases antibiotic resistance in bacteria.

### RNA and Protein Synthesis Inhibitors

Aminoglycosides are inhibitors of protein synthesis through interacting with the 30S ribosomal subunit, causing mRNA misreading (Davis, 1987). Besides its protein synthesis inhibiting effect, gentamicin also interacts with the outer membrane of Gram-negative bacteria through its cationic charge, and disrupts the LPS network by displacing Ca^2+^ and Mg^2+^ (Kadurugamuwa et al., 1993). The outer membrane is more likely to protrude, with OMV formation, as reported by Kadurugamuwa et al. for *P. aeruginosa* incubated with gentamicin (Kadurugamuwa et al., 1993). Additionally, these membrane vesicles carry greater amounts of DNA and cytoplasmic enzymes such as alkaline phosphatase in comparison with normal pinched-off OMVs. It has also been argued that the bacteria transfer virulence factors through OMVs (Kadurugamuwa and Beveridge, 1995; Ciofu et al., 2000). For instance, *Beta*-lactamase is packed into *P. aeruginosa* OMVs under the effect of benzylpenicillin (Ciofu et al., 2000). Subinhibitory concentrations of gentamicin increase both the production rate and size of the OMVs released from *Acinetobacter baylyi*. Moreover, these vesicles contain a higher amount of DNA compared with other stress-induced vesicles, and even if they contain the same amount of DNA, vesicles produced after gentamicin exposure are more efficient at transferring plasmid DNA (Fulsundar et al., 2014). Located gentamicin on the surface of the OMVs, gentamicin facilitates interbacterial interactions as well as membrane fusion (Kadurugamuwa and Beveridge, 1996; Schooling and Beveridge, 2006).

Macrolides, chloramphenicol, and tetracyclines are inhibitors of protein synthesis (Ruiz and Rámirez-Ronda, 1990). Only a limited number of studies have been allocated to the effect of these groups of antibiotics on changing membrane vesiculation patterns. The available results showed that they exert neither a significant inducing effect on membrane vesicle production nor on the level of virulence factors carried by membrane vesicles (Bauwens et al., 2017; Volgers et al., 2017; Yun et al., 2018). In contrast to aminoglycosides, these groups of antibiotics do not directly affect membranes, and do not disrupt membrane stability. Hence, it is plausible that these antibiotics are not direct inducers of membrane vesiculation. The lower the membrane vesiculation-inducing effect, the lower the likelihood that bacteria will develop resistance against an antibiotic. Although, membrane vesiculation is not the only mechanism in bacteria to being resistant against antibiotics.

### DNA Synthesis Inhibitors and Genotoxic Agents

Fluoroquinolones can induce structural changes in DNA by inhibiting DNA gyrase or topoisomerase IV (Hawkey, 2003). As this group of antibiotics causes double-strand breaks in DNA, it is believed that activation of the SOS pathway establishes considerable persistence against fluoroquinolones (Dörr et al., 2009). Maredia et al. observed that activation of the SOS pathway following *P. aeruginosa* exposure to ciprofloxacin has an increasing effect on OMV production in both the wild-type strain and strains mutant for LexA, the protein responsible for promoting the SOS pathway (Maredia et al., 2012). Moreover, in an old, heterogeneous *E. coli* biofilm, the SOS pathway induced by starvation or partial amino acid depletion is responsible for the observed tolerance to quinolone. Given that starved bacteria are more likely to release OMVs, it is likely that OMV production may also have a role in the increased tolerance against this antibiotic (Bernier et al., 2013; Chan et al., 2017). In *Stenotrophomonas maltophilia*, treatment with ciprofloxacin stimulates the production of OMVs as well as OIMVs. Additionally, some of these membrane vesicles are enriched with various stress response proteins and virulence factors, and some of the larger membrane vesicles have fimbriae on the surface. It is believed that these membrane vesicles can transfer virulence factors to other nearby bacteria, as well as induce vesiculation in these bacteria, even across species (Devos et al., 2017). In enterohemorrhagic *E. coli* (EHEC), treatment with ciprofloxacin can successfully increase OMV production, an effect that is accompanied by the induction of prophages, including that encoding Shiga toxin 2a (Bauwens et al., 2017). In *Francisella tularensis*, a ciprofloxacin-induced increase in OMV production enhances the tendency for biofilm formation (Siebert et al., 2019).

Genotoxic agents such as mitomycin C can greatly increase membrane vesicle production via the promotion of RecA-dependent stress responses, and activation of this pathway can trigger prophage activities. Prophage-encoded endolysin can damage the peptidoglycan layer through the activation of the holing–endolysin complex, which is followed by disruption of membrane integrity in both Gram-positive and Gram-negative bacteria (Turnbull et al., 2016; Toyofuku et al., 2017).

### Cell Wall and Cell Wall Synthesis Disruptors

**β-lactams** are putative inhibitors of cell wall synthesis. They are broad-spectrum antibiotics with highly specific targets in the bacterial domain, i.e., penicillin-binding proteins (PBPs) (Waxman and Jack, 1983). However, bacteria have established a high-level resistance to this group of antibiotics. Membrane vesicle production plays an important role in bacterial tolerance against β-lactams. Koning et al. (2013) observed that, under the effect of cephalosporin, *Acinetobacter baumannii* generated a higher number of membrane vesicles compare to the non-treated bacteria. They also observed that these vesicles originated primarily from the septum between dividing cells. Moreover, in addition to the outer membrane, most of the released vesicles also contained peptidoglycans as well as some inner membrane, indicating that they were OIMVs. Clinical *A. baumannii* strains establish carbapenem-resistant communities by transferring the gene encoding carbapenem hydrolyzing enzyme through OMV production (Rumbo et al., 2011). Besides the OMV-mediated horizontal gene transfer, *A. baumannii* OMVs carry various virulence factors, most important of which are active β-lactamase and OMP A. The latter is delivered to the host cell via OMVs that enter the cell through cholesterol-enriched domains (Jin et al., 2011). When treated with imipenem, the OMVs of *A. baumannii* showed higher toxicity against epithelial cells; interestingly, this resulted from the selective packing of virulence factors, such as periplasmic protease, into the OMVs (Yun et al., 2018). In EHEC, treatment with fosfomycin and meropenem increased OMV production; however, because the Shiga toxin 2a prophage is not induced by these groups of antibiotics, these vesicles did not show higher toxicity following treatment with cell wall synthesis inhibitors (Bauwens et al., 2017). The disruption of peptidoglycan synthesis, as well as the disturbed balance between the amount of peptidoglycan in the cytoplasm and outer membrane, may be the key reasons for the β-lactams-induced vesiculation (Deatherage et al., 2009; Koning et al., 2013). In respiratory tract infections, *Moraxella catarrhalis* and *Haemophilus influenzae* shed OMVs containing periplasmic β-lactamase, which is transferred to amoxicillin-susceptible species and other pathogens in a co-infection, such as Group A Streptococci. Moreover, β-lactamase in OMVs is sheltered against antibody-mediated immunity, thereby helping the infection become chronic (Schaar et al., 2013, 2014). The acquisition of β-lactamase via OMVs has also been seen in human gut microbiota, where *Bacteroides* species produce a cephalosporinase and transfer it to other commensals such as Bifidobacterium and gut pathogens such as *Salmonella typhi* (Stentz et al., 2015). In *S. aureus*, thermolabile, active β-lactamase is effectively protected against host proteases in OMVs (Lee et al., 2013). Devos et al. (2015, 2016) studied imipenem-induced hypervesiculation in *S. maltophilia* and observed that the quorum-sensing signal in this bacterium can effectively induce the production of OMVs, as well as virulence factors such as β-lactamase and biofilm formation-related proteins (Devos et al., 2015). In summary, β-lactams trigger membrane vesiculation, increasing bacterial resistance against these antibiotics. Membrane vesicles contribute significantly to horizontal gene transferring, packaging and transfer of virulence factors, and protecting virulence factors such as β-lactamases and hydrolases against antibacterial treatments. All the above examples show that membrane vesicles protect bacteria against cell wall synthesis inhibitors.

Polymyxins are cyclic cationic antimicrobial peptides that act by perturbing outer membrane integrity and changing membrane permeability. Mannig and Kuehn investigated the protective role of OMVs for bacteria in adsorbing and neutralizing the threats of outer membrane-interacting agents such as polymyxin B, colistin, and the effector components of the immune system against pathogenic bacteria. They revealed that, in the presence of membrane-interacting antimicrobials, *E. coli* survive by producing OMVs; however, in the presence of OMVs, the bacteria could not develop a resistance phenotype because the active doses of the antimicrobial agents decreased owing to interactions with OMVs (Manning and Kuehn, 2011). The protective role of OMVs shed by *M. catarrhalis* to keep the bacteria against antibiotics is not only associated with neighboring bacteria during respiratory infection, but also passively protects *Candida albicans* from cationic peptides and serum complement by sequestering them away from the environment of the fungus (Roszkowiak et al., 2019). Moreover, the inductive effect of this group of antibiotics on OMV production has also been observed in *P. aeruginosa* PA14, the clinical strain of *A. baumannii*, and *C. jejuni*. Indeed, in these bacteria, presenting with a hypervesiculation phenotype promotes bacterial survival in the presence of polymyxins (MacDonald and Kuehn, 2013; Yun et al., 2018).

## Chemical Agents

Chemical treatments against bacteria can be grouped into various categories, including heavy metals, organic solvents, and chelators. Exposure to these agents can disrupt membrane integrity, while their accumulation in cells perturbs the physiological activities of cell components. Even in solvent-tolerant bacteria that are highly tolerant against the harms of organic solvents, overexpression of SOS pathway genes increases their rescue rates against these toxins (Aonoa, 1998; Sardessai and Bhosle, 2002). The toxicity caused by heavy metals such as Pb (II) and Hg (II) is mainly followed by the induction of oxidative stress (Hussein and Joo, 2013).

The toxicity associated with organic solvents has been extensively reviewed, including bacterial responses to the presence of toxic organic solvents at the membrane, bacterial envelope, and cellular levels, as well as their underlying mechanisms. The most studied mechanism involves changes in membrane lipid composition. At the membrane level, as observed with high-temperature stress, the fast, adaptive response is also applicable in encountering toxic organic solvents (Okuyama et al., 1991; Heipieper et al., 1992). To date, *cis*-*trans* isomerization has been shown to be employed as an adaptive response to stress in strains of all known *Pseudomonas* spp. (Heipieper et al., 1992; Molina-Santiago et al., 2017), *Vibrio* spp. (Okuyama et al., 1991), *Methylococcus capsulatus* (Löffler et al., 2010), *Alcanivorax borkumensis* (Naether et al., 2013), and *Colwellia psychrerythraea* (Hashimoto et al., 2015). *Cis-trans* isomerization contributes to the high adaptability, particularly of *Pseudomonas* and *Vibrio* species, to diverse ecosystems, giving them high durability in adverse environmental situations. Nonetheless, this mechanism is not applicable to all bacteria (Tan et al., 2016).

Toxic and mostly hydrophobic solvents tend to localize in the membrane, and changing the fatty acid composition through reducing membrane lipophilicity can be beneficial for bacteria (Weber and de Bont, 1996). At the envelope level, Gram-negative bacteria benefit from modifying the LPS composition. For instance, *P. putida* changes the B-band/A-band ratio in favor of B-band in the presence of hydrophobic solvents like o-xylene. This change not only decreases membrane hydrophobicity, but can also lead to the discharge of OMVs from the B-band area. It is plausible that the treatment agents could be packaged inside the vesicles, thereby helping bacteria to withstand the chemical treatment (Pinkart et al., 1996; Baumgarten et al., 2012a; Eberlein et al., 2019). After the membrane blebbing from the B-band area, the accumulation of remain A-band LPS renders the surface of the bacteria more hydrophobic, and higher hydrophobicity results in a higher propensity for biofilm production (Pasmore et al., 2001; de Carvalho et al., 2009; Baumgarten et al., 2012a). This indicates that forming a biofilm may be another consequence of the bacterial defense mechanism, or a direct approach utilized by bacteria to survive in the presence of organic solvents. The contents of OMVs also help the construction of the biofilm matrix (Aono and Kobayashi, 1997; Eberlein et al., 2018). The production of hydrophobic signaling molecules in the presence of organic solvents is another proposed mechanism, which helps the outer membrane become more lipophilic herby, the OMV discharging from the outer membrane enhances (Baumgarten et al., 2012b; Eberlein et al., 2018). However, Baumgarten et al. believe that organic compounds, such as chlorinated phenols, hinder the production of quorum-sensing signaling molecules, and harm cells by preventing OMV generation (Baumgarten et al., 2012b). The bacterial response to the presence of organic solvents or toxic chemicals contains some back reactions of the adaptation responses. For example, the Cti protein, which catalyzes the *cis-trans* isomerization reaction in solvent-tolerant as well as other bacteria, undergoes posttranslational modifications under organic solvent-induced stress (Heipieper et al., 2003). Organic solvents can trigger the expression of chaperonins, the activities of which are in balance with membrane vesicle production (Yang et al., 2020).

Chelators also have toxic effects against bacteria since they can trigger the depletion of cations that stabilize the membrane structure (Che et al., 2009). EDTA is known to be a membrane perturbing agent through its ability to sequester Mg^2+^ and Ca^2+^ from the cell wall, and partially reduce the interaction between the cell wall and cytoplasmic membrane; consequently, EDTA can boost membrane vesicle production (Matsushita et al., 1978; Finnegan and Percival, 2015). However, contradictory effects have also been observed in the fungus *Cryptococcus neoformans* (Robertson et al., 2012). McDaniel et al. (2016) observed that a *P. aeruginosa* strain mutant for B-band lipopolysaccharide production was highly liable to EDTA effects. Given the crucial importance of B-band LPS in OMV discharge, it is plausible that membrane vesicle production may be a defensive mechanism against EDTA-induced stress.

The amino acid glycine is an inhibitor of cell wall synthesis that promotes spheroplast formation, leading to bacterial lysis with prolonged incubation. Glycine can disrupt the transpeptidation step of bacterial cell wall synthesis by replacing D-alanine; thus, the bacterial cell wall cannot be properly formed in the presence of glycine (Hishinuma et al., 1969). Moreover, accumulation of the building blocks of the bacterial cell wall—i.e., *N*-acetyl glucosamine and *N*-acetylmuramic acid—and parts of the incomplete cell wall perturb the balance between intra- and extracellular cell wall components, which induces membrane vesicle formation (Hirayama and Nakao, 2020). Hirayama et al. used glycine to induce membrane vesiculation in the probiotic *E. coli* strain Nissle 1917 and observed deformity and quasi-lysis during logarithmic and stationary growth phases, respectively, resulting in a 6–8-times higher rate of vesicle expulsion; moreover, membrane vesicles produced in the stationary phase were larger and more deformed than those in the exponential phase. The authors hypothesized that vesicles in the exponential phase originate from membrane blebbing whereas those in the stationary phase mainly arise from explosive cell lysis (Deatherage et al., 2009; Hirayama and Nakao, 2020).

## Phages and Prophages

Bacterial genomes possess a repertoire of prophage gene clusters, the expression of which can be induced under the effect of various stressors in favor of rescuing the bacterial population. According to the most recent studies on *B. subtilis* and *P. aeruginosa*, peptidoglycan lysis via phage-derived endolysin can trigger explosive cell lysis. Furthermore, some parts of the broken down membrane come together and create membrane vesicles. Hence, prophage activators can act as inducers of membrane burst-induced membrane vesicle discharge (Toyofuku et al., 2014; Turnbull et al., 2016). Numerous studies have investigated the various stresses that promote prophage activation in both Gram-positive and Gram-negative bacteria; however, the key mechanism downstream of prophage activation is the induction of the SOS pathway. Oxidative stress, as well as anoxia (Fang et al., 2017), UV radiation (Kageyama et al., 1979), iron depletion (Binnenkade et al., 2014), antibiotic treatment (especially genotoxins) (Beaber et al., 2004), and unfavorable pH (Bamford et al., 1987) are among the well-characterized effectors of prophage activation. In addition to lysis proteins, some prophages also encode bacterial toxins such as Shiga toxin 2a. Although ciprofloxacin and mitomycin C can both induce Shiga toxin 2a expression in EHEC strains, treatment with cell wall synthesis inhibitors did not show prophage activating effects (Bauwens et al., 2017). The prophage inducing activities of ciprofloxacin have also been evaluated in *S. maltophilia* and *P. aeruginosa*, *Vibrio cholera*, *S. aureus*, and *S. enterica* (Beaber et al., 2004; Úbeda et al., 2005; Bearson and Brunelle, 2015; Langan et al., 2015). Notably, as prophage activation is more likely to be under the control of the SOS pathway, and its activation works in parallel with cellular stress responses, the released membrane vesicles are enriched with stress response proteins (Devos et al., 2017). Imipenem causes differential expression of the proteins related to cryptic phage islands in *A. baumannii*, while the overall amount of phage proteins packed in OMVs decreases by approximately 40 percent when compared with those derived from untreated cells (Yun et al., 2018). Another comparative study on cytoplasmic membrane vesicle formation in *S. aureus* revealed that phage-induced membrane vesicles carry a significantly higher amount of DNA compared with the antibiotic-induced membrane vesicles in phage-devoid strains (Bearson and Brunelle, 2015). Hence, prophage-induced membrane vesicles are effective at horizontal gene transfer (Andreoni et al., 2019; Crispim et al., 2019). This capacity of prophages to induce membrane vesicle production has also been acknowledged in studies focusing on the immunopathogenicity of bacteria, where the membrane vesicles seem to help protect the bacteria from the immune system via various mechanisms (Andreoni et al., 2019; Wahl et al., 2019). Moreover, using *E. coli* as a model, Manning et al. observed that the bacteria benefit from OMV production to survive against bacteriophage T4. Given that OMVs act as a cellular decoy, OMV-bacteriophage T4 interaction significantly reduces bacteriophage infectivity (Manning and Kuehn, 2011). A protective role of membrane vesicles for the bacteria producing them has also been proposed for oceanic bacteria as they were found to release membrane vesicles containing a phage receptor (Biller et al., 2014).

## Host–Bacteria Interactions

**FIGURE 4:**
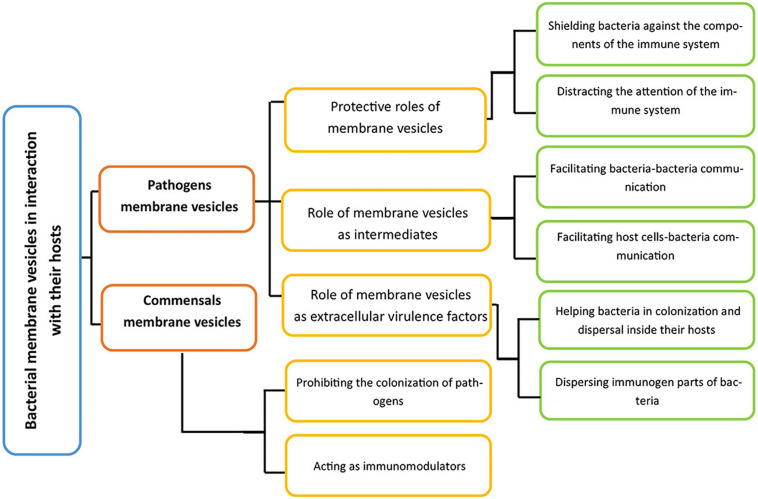
A general perspective of ways by which bacteria interact with their hosts via their membrane vesicles. Membrane vesicles inside the host could originate from pathogens and commensal. For the former group, there are three roles, and the latter group also has two crucial activities inside their hosts.

Membrane vesicles not only help bacterial become adapted to various abiotic factors of their environment (Figure 3), they also regulate bacterial–host interactions. The role of membrane vesicle as an intermediate for bacteria and host interactions could be investigated through various perspectives (Figure 4).

The evolutionary roots of a requirement for membrane vesicle production by pathogenic bacteria inside their hosts, in a significantly higher amount when compared with *in vitro* models, is not completely clear, as their production has both advantages and disadvantages for the bacterial community. A very simple explanation for the occurrence of the hypervesiculation phenotype by pathogenic bacteria during infection is that the hostile environment of the host organs is a source of various stresses, which forces the bacteria to produce a higher amount of membrane vesicles (Tashiro et al., 2009; Schwechheimer and Kuehn, 2015).

### Host–Pathogen Interaction

As depicted in Figure 4, the interactions between pathogen-derived membrane vesicles and their host can be split into three crucial parts, depending on the role of the vesicles:

(I) The protective role of membrane vesicles for pathogens: Irrespective of the biogenesis pathway, membrane vesicles comprise both a part of the bacterial membrane as well as other components. The carrying of the bacterial antigen by membrane vesicles distracts the attention of the host immune system (Kulp and Kuehn, 2010). The defensive components of the immune system lose their effectiveness against bacterial infection as a part of its components is in interaction with membrane vesicles mimicking the bacterial envelopes (Ellis and Kuehn, 2010; MacDonald and Kuehn, 2012). Additionally, since membrane vesicles have bacterial ligand molecules, they can enter host immunity cells, and disrupt their effective response to the infection. The mechanisms of their entry have been extensively reviewed elsewhere (Ellis and Kuehn, 2010; O’donoghue and Krachler, 2016).

(II) Membrane vesicles as intermediates to facilitate the interaction of pathogens with their hosts: In Gram-negative bacteria, OMVs are considered to be an effective secretion system as they can transfer numerous molecules regardless of their physicochemical features. Thus, they can act as a delivery system between bacteria as well as a communication system between bacteria and their eukaryotic hosts (Guerrero-Mandujano et al., 2017). Membrane vesicles also function as a delivery system in Gram-positive bacteria, effectively transferring virulence factors, such as those that suppress the host immune system (MacDonald and Kuehn, 2012). Moreover, bacterial membrane vesicles carrying antibiotics can exert cytotoxic effects on nearby eukaryotic cells (Kadurugamuwa and Beveridge, 1998).

(III) Membrane vesicles as extracellular virulence factors: Not only do the membrane vesicles convey bacterial virulence agents to their host cells and help the bacteria release these molecules into their extracellular milieu, they also benefit bacteria by protecting their virulence components against host immune systems (Kuehn and Kesty, 2005; MacDonald and Kuehn, 2012). Membrane vesicles can help pathogens with colonization and dispersal. For instance, membrane vesicles greatly contribute to biofilm formation as well as its stabilization, and pathogens benefit from biofilm formation through the formation of a secluded area to protect their community from the host immune system (Kuehn and Kesty, 2005; MacDonald and Kuehn, 2013). Additionally, membrane vesicles can facilitate bacterial penetration into host tissues without being exposed to the host immune system on the one hand, while on the other, repetitive exposure of the immune system to the highly immunogenic components on membrane vesicles leads to the activation of tolerance mechanism against the pathogen, allowing the bacteria to disperse (Chi et al., 2003; Elmi et al., 2016; Cecil et al., 2019).

The production of membrane vesicles is not always in favor of bacteria. For instance, in terms of host–pathogen interactions, membrane vesicles can be disadvantageous for bacteria as they possess significant immunogenic activities that can trigger both innate and adaptive immune responses. Consequently, these features make the vesicles suitable candidates as targets for vaccines and adjuvant formulations. The immunomodulatory effects of membrane vesicles have been comprehensively reviewed (Ellis and Kuehn, 2010; Beceiro et al., 2013; Cecil et al., 2019).

### Host–Commensal Interaction

Hosts benefit from the membrane vesicles of their commensals in various ways, the most important of which are:

(I) Inhibition of pathogen colonization: By promoting the successful colonization and dispersal of commensals, the membrane vesicles of commensals compete with those of the pathogens and prevent them from becoming dominant communities inside the host tissues (Macia et al., 2020).

(II) Regulation of host immune system responses: The immunomodulatory roles of commensal membrane vesicles are highly significant. Mounting evidence has indicated that they exert anti-inflammatory effects, while the membrane vesicles of probiotics such as *Bacteroidetes* spp., *Lactobacillus* spp., *Bifidobacterium* spp., commensal *E. coli* spp., and *Akkermansia muciniphila* boost mucosal tract immunity (Brown et al., 2015; Cecil et al., 2019; Caruana and Walper, 2020). These properties of membrane vesicles have been applied in the treatment of chronic diseases such as inflammatory bowel disease and ulcers caused by *H. pylori* (Uccello et al., 2012; Molina-Tijeras et al., 2019). The interaction between the human host and microbiota has been reviewed by Cecil et al. (2019). Additionally, Macia et al. and Caruana et al. have reviewed the immunomodulatory roles of the membrane vesicles of commensals from a general perspective (Caruana and Walper, 2020; Macia et al., 2020). Importantly, membrane vesicles of commensals can also be disadvantageous to their hosts as they can transfer virulence factors, such as antibiotic resistance genes, to pathogens (Stentz et al., 2015).

## Conclusion

Bacterial membrane vesicles are important elements of the bacterial surface, and numerous studies have shed light on the physiological and ecological features of bacterial membrane vesicles, as well as their community-dependent functions. Moreover, accumulating evidence has indicated that membrane vesicles endow bacteria with an extraordinary capacity to survive in stressful conditions, and are considered to be a potent bacterial innate immune element. For pathogens, membrane vesicles exert protective effects against antibacterial agents, and controlling or inhibiting the production of membrane vesicles will prove beneficial in the battle against pathogenic bacteria. In this review, a comprehensive perspective concerning the stressors that alter pathogen membrane vesiculation patterns have been presented. The results of studies undertaken to date have shown that interfering with membrane vesiculation not only decreases bacterial viability, but can also effectively control the virulence of bacteria and reduce the acute consequences of bacterial infections as well. In this context, components of pathways involved in membrane vesiculation are potentially promising candidates as targets for antibacterial treatments. Hence, further studies on inducers of membrane vesiculation, the contents of membrane vesicles, and ways to control membrane vesiculation will lead to the development of new antibacterial treatments. Despite the numerous studies on the various functions of membrane vesicles, the approach of controlling membrane vesicle production for addressing the antibiotic resistance problem is mostly a neglected area. Hence, to introduce this approach as a strategy against highly resistant pathogens, more research needs to dedicate to the ways of controlling membrane vesiculation and the contents of vesicles.

## Author Contributions

NM designed and wrote the first draft. NM and M-PM-L finalized the manuscript. Both authors contributed to the article and approved the submitted version.

## Conflict of Interest

The authors declare that the research was conducted in the absence of any commercial or financial relationships that could be construed as a potential conflict of interest.
