# Supplementary material for: Membrane Vesicle Production as a Bacterial Defense Against Stress
Source: Front Microbiol. 2020 Dec 9;11:600221. doi: 10.3389/fmicb.2020.600221 (PMC7755613; doi:10.3389/fmicb.2020.600221)
Supplement: Supplementary file 1 [file Table_1.docx]

Supplementary Material

Membrane Vesicle Production as a Bacterial Defense Against Stress

Negar Mozaheb^1^, Marie-Paule Mingeot-Leclercq ^1*^

^1^ Université catholique de Louvain (UCL), Louvain Drug Research Institute (LDRI), Cellular & Molecular Pharmacology Unit (FACM), 1200 Brussels, Belgium

*** Correspondence:**Marie-Paule Mingeot-Leclercq
marie-paule.mingeot@uclouvain.be

Supplementary Table 1. List of studies allocated to the membrane vesicles. The information of the table summarizes the studies in terms of the main approach of studies, assessed effects, and conclusions.

| ***Effectors*** | | ***Microorganism*** | ***Main Approaches*** | ***Assessed Effects*** | ***Other Conclusions*** | ***Ref.*** |
| --- | --- | --- | --- | --- | --- | --- |
| **Environmental Factor** | **Oxidative Stress** | C. jejuni | Activation of the general stress response | -Increased OMV numbers  -Alteration of OMVs’ composition  -Role of chaperonins in OMV production rate | - Presence of Flagel proteins in OMVs  - OMVs act as a virulence factor | (1) |
|  |  | P. aeruginosa PAO1 | - Activation of the SOS response  - Induction of nitrogen-dependent respiration | - Production of F and R-pyocins  - Increased OMV numbers | -Presence of prophage components in the OMVs | (2) |
|  |  | E. coli | - Mutation in oxyR gene | - Increased OMV numbers | -Effect of disrupted membrane on OMV production | (3) |
|  |  | N. meningitides | - Cysteine depletion  - Increasing the dissolved oxygen in the culture of mutant strains for peptidoglycan anchored protein (∆rmpM) | - Increased OMV numbers  - Decreased the growth rate of ∆rmpM  - The presence of SOS response elements in OMVs | - Activation of SOS response pathway during cysteine depletion | (4), (5) |
|  |  | *P. aeruginosa* | -Ectopic expression of the main bacterial chaperonins  -Tracking the O-antigen structural change | -The higher chaperonins expression the lower amount of OMV  - B-band polysaccharide is required for membrane blebbing | - Misfolded proteins in periplasm induce OMV production  -OMVs are originated from high curvature area of OM | (6), (7), (8) |
| **Environmental Factor** | **Oxidative Stress** | *C. rodentium* | -Perturbing membrane integrity by creating mutants for two-component system (PmrAB/C) activating by iron | - High concentration of iron induces oxidative stress | - High concentration of iron disturbs membrane integrity | (9) |
|  |  | *L. interrogans* | -Increasing the concentration of free iron  -Inducing mutation in metalloprotease *Htpx* homolog | -Membrane vesicle production increase in the presence of soluble iron  -Membrane and membrane vesicles contribute to passive nucleation of metals | - *Htpx and its* homologs could regulate the stress response genes | (10) |
|  | **UV exposure** | Fresh water bacteria | -UVR exposure (UVA+UVB) | -Increased membrane vesicle production |  | (11) |
|  |  | *C. raciborskӀ* | -UV exposure  (UVA+UVB) | -Increased membrane vesicle production  -Increase exopolysaccharide in produced membrane vesicle | -Exposure of phosphatidylserine is a manifestation of hypervesiculation phenotype | (12) |
|  | **Nutrient deprivation** | *N. meningitides* | -Cysteine depletion  -Lysine depletion | - General stress response activation  -Increased OMV production | - Cysteine, lysine, and iron depletion make oxidative stress | (4, 13) |
|  |  | ETEC | Iron limitation | -Increased OMV production | - The presence of the general stress response proteins like UvrA and UpsF in the OMVs | (14) |
|  |  | *P. gingivalis* | -Iron limitation | -OMV production carrying HmuY, heme-binding activity, increased | - Increase of HmuY in media | (15) |
| **Environmental Factor** | **Nutrient deprivation** | *N. meningitides* | -Sulfate depletion  -Determining the role of VacJ/Yrb ABC (ATP-binding cassette) | -Increased OMV numbers  -Activation of the SOS response pathway  -Perturbation of the outer membrane asymmetry | -Sulfate depletion cause oxidative stress  -Under the oxidative stress, VacJ/Yrb ABC transporter is downregulated | (16) |
|  |  | *S. enterica* | -Mg^2+^ depletion | - Activation of the PhoPQ system  -Increase OMV production | -Lipid A deacetylation causes an increase in the high curvature area of the membrane | (17) |
|  |  | *P. aeruginosa* | -Mg2+ depletion | -Increased OMV numbers  -Activation of the SOS response pathway |  | (18) |
|  |  | *P. aeruginosa* | -Induced iron-depletion via knock-outing the putative systems for iron uptake | -An effective iron acquisition system based on type VӀ secretion system and OMVs containing PQS | -The iron-PQS complexes on the OMV surfaces could transfer iron to the other cells possessing the receptors of these complexes ligands’ | (19) |
|  |  | *V. cholera* | -Hyper-vesiculation mutants  -Mutants in membrane asymmetry maintaining system  -Polymyxin B  -High bile-salt concentration | -Role of membrane asymmetry maintaining system in hypervesiculation  -Role of hyper-vesiculation in cope with starvation stress, antimicrobial cations, and bile-salt | -LPS modification and hypervesiculation help pathogens with faster adaption and colonization in their hosts | (20) |
| **Environmental Factor** | **Nutrient deprivation** | *M. tuberculosis* | -Induced iron-depletion  -Mutant creation for siderophores | -Increased membrane vesicle production carrying mycobactin | -Mutant strain for mycobactin production is not able to grow in minimal condition without an exogenous source for free iron or siderophores | (21) |
|  | **pH** | *F. tularensis* (clinical strain) | -Mimicking the environment inside the mammalian host (low pH) | -Membrane vesicles are tubular  - Increased OMV production | -The protein content of OMVs alteration in various stress condition | (22) |
|  |  | *S. enterica* serovar Typhimurium (*S. Typhimurium*)/ S. enterica | -Analysis of OMV’s RNA content under low pH condition | -The presence of sRNA responsible for *E. coli* resistance against low pH  - Increased OMV production  -Induced changes in the outer membrane under low pH condition | -The RNA content of OMVs play various roles  - Activation of PhoP/Q and PmrA/B two-component systems are responsible for inducing outer membrane changes | (23-25) |
|  |  | *V. fischeri* | -Culturing the bacteria at acidic or basic pH | - Increased OMVs carrying ompU  -The higher OMV, the higher the symbiotic signal production | - Various environmental conditions change the protein content of OMVs | (26) |
|  | **Temperature** | *E. coli* | -Increasing the temperature up to 55 C˚ for 10 minutes | - Increased OMV numbers |  | (27) |
|  |  | *P. aeruginosa* PAO1 | -Increasing the temperature to 45 C˚  - Modifying the strains for developing rough phenotype (without O-antigen) | - Increased OMV numbers  -Rough strains produce larger OMVs after the heat treatment | -O-antigen is necessary for OMV production induced by the high temperature | (28, 29), (30) |
| **Environmental Factor** | **Temperature** | *E. coli, P. aeruginosa,* *C. jejuni* | - Ectopic expression of the envelope stress response components | -Increased OMV production after heat treatment |  | (31-34) |
|  |  | *P. aeruginosa PAO1* | -Inducing PQS production via heat shock | - Mostly no change in OMV production | - PQS can induce OMV production via interacting with membrane  -Under stress, PQS is not necessary for OMV production | (35), (6) |
|  |  | *P. putida* DOT-T1E | -Heat treatment in 55 C˚ for 10 minutes | -Increased OMV numbers  -changed OMV contents | - High temperature induces the production of OMVs containing more saturated fatty acids  - OMV production increases bacterial surface hydrophobicity  -Bacteria tend to produce a higher amount of biofilm | (28, 36) |
|  | **Osmotic pressure, Desiccation, and Hydration** | *F. tularensis* | -Heat treatment  -Cold Treatment | -Increased vesiculation under the effect of both treatments  - Heat-induced vesicles carry the enzymes involved in the bacterial envelope biosynthesis and metabolism | -Low pH, as well as high temperature, effectively induce the production of membrane vesicles | (22) |
|  |  | *P. putida* DOT-T1E | -High-saline medium (2 Molar) | -Increased OMV numbers  -changed OMV contents | - The high temperature induces the production of OMVs containing more saturated fatty acids  - OMV production increases bacterial surface hydrophobicity | [20] |
| **Environmental Factor** | **Osmotic pressure, Desiccation, and Hydration** | *L. monocytogenes* | - High-saline medium | - An increased concentration of misfolded protein  -Increased OMV production  - Decreased amount of misfolded protein after HtrA activation | -OMV production and the periplasmic chaperonins act in two opposed ways | (32) |
|  |  | *L. monocytogenes* | -Induced high osmotic pressure using KCl and NaCl | -Showing Hyper-vesiculation phenotype helps the bacteria with transferring the compatible solutes to each other | -NaCl has more potency for inducing vesiculation | (37) |
|  |  | *E. coli* | -Determining the role of osmotic pressure-response elements (OmpR) by mutant fabrication (∆*ompR*) | -OmpR does not directly take part in OMV production |  | (38) |
|  |  | *E. coli* | -Induced osmotic pressure by and dehydration condition using glycerol/water solution and freely permeant polyol respectively  -High-speed rehydration | -Cell death membrane vesiculation under osmotic, desiccation, and rehydration stresses  -Membrane lipid phase transition |  | (39) |
| **Antibiotics** | **Aminoglycosides** | *P. aeruginosa* | -Gentamicin treatment | -Increased OMV production  -Virulence factors are packed in OMVs after the treatment with gentamicin | -Replacement of the antibiotic with Ca2+ and Mg2+ in the outer membrane causes membrane bulging | (40), (41, 42) |
| **Antibiotics** | **Aminoglycosides** | *A. baylyi* | -Treatment with sub-inhibitory concentrations of gentamicin | -Increased OMVs carrying virulence factor production | -These vesicles are successful in transferring DNA | (43) |
|  | **Macrolides. Tetracyclines** | *E. coli,* *M. catarrhalis,* and *A. baumannii* (clinical strain) | Treatment with azithromycin and tetracycline | -Neither change in membrane vesicle production nor in the cytotoxicity of the vesicles | -Inhibitors of cell-wall synthesis act more effectively in inducing membrane vesicle production than the inhibitors of protein synthesis | (44-46) |
|  | **Fluoroquinolones** | *P. aeruginosa*, *E. coli* | -Treatment with ciprofloxacin | -Increased OMV production  -Induction of SOS response pathway | -Ciprofloxacin induces the SOS response pathway  -Increased toleration against the antibiotic especially in biofilm | (18),(47) |
|  |  | *S. maltophilia* | -Treatment with ciprofloxacin | -Increased OMV and OIMV production  -Some of the vesicles have fimbriae compartments | -Vesicles are the capable intermediate for transferring the virulence factors | (48) |
|  |  | EHEC | -Treatment with ciprofloxacin | -Increased OMV number  -Activation of prophage harboring shi-ga toxin 2a  -Production of Shiga toxin- associated OMVs | -Antibiotic treatment increases the toxicity of OMVs in EHEC | (44) |
| **Antibiotics** | **Fluoroquinolones** | *F. tularensis* | -Treatment with ciprofloxacin | -Increased OMV number  -Increased tendency for biofilm formation |  | (49) |
|  | **Genotoxins** | *B. subtilis, P. aeruginosa* | -Treatment with mitomycin C | -Increased OMV production | -Prophage activation  -Peptidoglycan lysis →  SOS response activation | (50, 51) |
|  | **ß-lactams** | *P. aeruginosa* | -Treatment with benzyl penicillin | -Increased OMV production packing ß-lactamase |  | (42) |
|  |  | *A. baumannii* | - Treatment with cephalosporin and imipenem | -Increased OMV and OIMV production  -Convey of ß-lactamase, carbapenemase and outer membrane protein with OMVs | -Membrane vesicles after antibiotic treatment show higher toxicity  -Membrane vesicles act as a suitable mediator for horizontal gene transfer | (52), (53), (46) |
|  |  | EHEC | -Treatment with meropenem and fosfomycin | -Increased OMV production | -The treatments are not able to activate prophage harboring Shiga toxin 2a | (44) |
|  |  | *M. catarrhalis, H. influenza* | No treatment | -OMVs transfer the ß-lactamase and ß-lactamase genes to Gram-positive and negative | -sheltered ß-lactamase in the OMVs is protected against host immune systems | (54, 55) |
| **Antibiotics** | **ß-lactams** | Bacteroides | Within gut microbiota/No treatment | -OMVs transfer cephalosporinase to other commensals and enteric pathogens like *S. typhi* |  | (56) |
|  |  | *S. aureus* | No treatment | -OMVs transfer the ß-lactamase | - The produced ß-lactamase is highly thermo-labile  -The ß-lactamase is protected from proteases by sheltering in OMVs | (57) |
|  |  | *S. maltophilia* | -Treatment with imipenem | -Increased OMV production |  | (58, 59) |
|  | **Polymyxins** | *E. coli* | -Treatment with polymyxin B, colistin | -Increased OMV number  -Antibiotic interaction with the OMVs → bacterial survival | -Bacteria will not be able to develop resistant community against the antibiotic | (60) |
|  |  | *P. aeruginosa PA14*, *A. baumannii* ,*C. jejuni* | -Treatment with polymyxin B | -Increased OMV production  -Bacterial survival |  | (1, 6, 46) |
|  |  | *M. catarrhalis* | -Using normally produced OMVs by *M.* *catarrhalis* | -Increased pathogenicity, and the survival rate of neighboring bacteria as well as *C. albicans* after the treatment with polymyxin B | -OMVs from *M.* *catarrhalis* passively protect the *C. albicans* from serum complement. Also, they help the yeast to keep its filamentous phenotype in the presence of cationic peptides and serum complement | (61) |
| **Chemicals** | **Organic Solvent** | *P. putida* | -Exposure to alkanol/ chlorophenols/ o-Xylene | -Change in the envelope composition  -Change in LPS composition  -Increase in OMV production  -Increase in biofilm formation potency of the stain | -Envelope changes have significant effects on bacteria toleration against organic solvents | (36, 62-65) |
|  | **EDTA** | *P. aeruginosa* | -Treatment with EDTA  -Mutant creation for B-band LPS production | -A decrease in OMV production  - Lower survival rate against EDTA |  | (66) |
|  |  | *P. aeruginosa* | -Treatment with EDTA | -A decrease in membrane stability | -Increase OMV production | (67-69) |
|  |  | *C. neoformans* | -Treatment with EDTA | -A decrease in membrane vesicle production  -A decrease in biofilm production | -Deficiency in the secretion of extracellular polysaccharide | (70) |
|  | **Glycine** | *E. coli* | - Treatment with glycine | -Induction of OMV production | -Treatment with glycine increases OMV formation *via* 2 separate biogenic mechanisms  - Induced and noninduced membrane vesicles have similar protein contents | (71) |
| **Phages** | **Prophage activation** | *B. subtilis* | -Treatment with genotoxins  -Fabrication of mutant strains for the gene in the SOS response pathway | -Increased membrane vesicle production triggered by endolysin activity  -Mutant strains fell short in membrane vesicle production | -The produced endolysin could trigger the membrane vesicle formation in neighboring cells | (50) |
|  |  | EHEC | -Treatment with Ciprofloxacin, meropenem, fosfomycin, polymyxin B, gentamicin, rifaximin, tigecycline, and azithromycin | -The effective treatment for induction of OMV production | -The activation of prophage harboring 2a Shiga toxin is responsible for cytotoxicity caused by OMVs | (44) |
|  |  | *S. aureus* | -Treatment of the S. aureus and its phage-devoid counterparts with mitomycin C, daptomycin ciprofloxacin, flucloxacillin, and ceftaroline | -Induced OMV production  -The failure of membrane vesicle formation after SOS stress response activation in phage –devoid strain |  | (72) |
|  |  | *P. aeruginosa* PAO1 | -Providing denitrifying condition | -Increased OMV production  -Induced pyocin activation | -Activation of the SOS response pathway under the denitrifying condition  -Increased PQS carrying OMVs  -Prophage activation under the denitrifying condition | (2) |
| **Phages** | **Prophage activation** | *P. aeruginosa* | -Applying genotoxins for inducing prophage activation | -The activation of lysins encoded by prophages induces explosive cell lysis  -Explosive cell lysis is a model for OMV production | -Explosive cell lysis promotes biofilm formation  -The activation of SOS pathway is prophage induction and responsible for explosive cell lysis | (51) |
|  |  | *D. alaskensis* | -Treatment with genotoxins | -Activation of prophages  -The presence of prophage genes in the OMVs released | -The genotoxic induced virus-like particles (VLPs) contribute to horizontal gene transfer | (73) |
|  |  | *A. baumannii* | -Treatment with Hydrogen peroxide, d-cycloserine, polymyxin B, and imipenem | - Differential expression of phage proteins correlated with antibiotic treatment | - Different in the toxicity of OMVs produced following by various antibiotic treatments | (46) |
|  |  | *S. maltophilia* | -Treatment with ciprofloxacin | - Induction of prophage activation  - Induction of membrane vesiculation | -Produced membrane vesicles are heterogeneous in terms of their shape and contents | (48) |
|  | **Phage interaction** | *E.coli* | -Polymyxin B and colistin treatment  -Hyper-vesiculating mutant creation | - Decreased efficacy of antimicrobial activity of antibiotics and T4 bacteriophage were |  | (60) |

References

1. Godlewska R, Klim J, Dębski J, Wyszyńska A, Łasica A. Influence of environmental and genetic factors on proteomic profiling of outer membrane vesicles from Campylobacter jejuni. *Pol J Microbiol* (2019) 68(2):255-61. doi: 10.33073/pjm-2019-027.

2. Toyofuku M, Zhou S, Sawada I, Takaya N, Uchiyama H, Nomura N. Membrane vesicle formation is associated with pyocin production under denitrifying conditions in P seudomonas aeruginosa PAO 1. *Environ Microbiol* (2014) 16(9):2927-38. doi: 10.1111/1462-2920.12260.

3. Kulp AJ, Sun B, Ai T, Manning AJ, Orench-Rivera N, Schmid AK, et al. Genome-wide assessment of outer membrane vesicle production in Escherichia coli. *PLoS One* (2015) 10(9). doi: 10.1371/journal.pone.0139200.

4. van de Waterbeemd B, Zomer G, van den IJssel J, van Keulen L, Eppink MH, van der Ley P, et al. Cysteine depletion causes oxidative stress and triggers outer membrane vesicle release by Neisseria meningitidis; implications for vaccine development. *PLoS One* (2013) 8(1). doi: 10.1371/journal.pone.0054314.

5. Gerritzen MJ, Maas RH, Van Den Ijssel J, Van Keulen L, Martens DE, Wijffels RH, et al. High dissolved oxygen tension triggers outer membrane vesicle formation by Neisseria meningitidis. *Microb Cell Fact* (2018) 17(1):1-10. doi: 10.1186/s12934-018-1007-7.

6. MacDonald IA, Kuehn MJ. Stress-induced outer membrane vesicle production by Pseudomonas aeruginosa. *J Bacteriol* (2013) 195(13):2971-81. doi: 10.1128/JB.02267-12.

7. Kadurugamuwa JL, Beveridge TJ. Bacteriolytic effect of membrane vesicles from Pseudomonas aeruginosa on other bacteria including pathogens: conceptually new antibiotics. *J Bacteriol* (1996) 178(10):2767-74. doi: 10.1128/jb.178.10.2767-2774.1996.

8. Sabra W, Lünsdorf H, Zeng A-P. Alterations in the formation of lipopolysaccharide and membrane vesicles on the surface of Pseudomonas aeruginosa PAO1 under oxygen stress conditions. *Microbiology* (2003) 149(10):2789-95. doi: 10.1099/mic.0.26443-0.

9. Sinha A, Nyongesa S, Viau C, Gruenheid S, Veyrier FJ, Le Moual H. Pmrc (EptA) and CptA negatively affect outer membrane vesicle production in Citrobacter rodentium. *J Bacteriol* (2019) 201(7):e00454-18. doi: 10.1128/JB.00454-18.

10. Henry R, Lo M, Khoo C, Zhang H, Boysen RI, Picardeau M, et al. Precipitation of iron on the surface of Leptospira interrogans is associated with mutation of the stress response metalloprotease HtpX. *Appl Environ Microbiol* (2013) 79(15):4653-60. doi: 10.1128/AEM.01097-13.

11. Gamalier JP, Silva TP, Zarantonello V, Dias FF, Melo RC. Increased production of outer membrane vesicles by cultured freshwater bacteria in response to ultraviolet radiation. *Microbiological research* (2017) 194:38-46. doi: 10.1016/j.micres.2016.08.002.

12. Zarantonello V, Silva TP, Noyma NP, Gamalier JP, Mello MM, Marinho MM, et al. The cyanobacterium Cylindrospermopsis raciborskii (CYRF-01) responds to environmental stresses with increased vesiculation detected at single-cell resolution. *Front Microbiol* (2018) 9:272. doi: 10.3389/fmicb.2018.00272.

13. Bishop D, Work E. An extracellular glycolipid produced by Escherichia coli grown under lysine-limiting conditions. *Biochem* (1965) 96(2):567. doi: 10.1042/bj0960567.

14. Chan KW, Shone C, Hesp JR. Antibiotics and iron‐limiting conditions and their effect on the production and composition of outer membrane vesicles secreted from clinical isolates of extraintestinal pathogenic E. coli. *Proteomics Clin Appl* (2017) 11(1-2):1600091. doi: 10.1002/prca.201600091.

15. Olczak T, Wójtowicz H, Ciuraszkiewicz J, Olczak M. Species specificity, surface exposure, protein expression, immunogenicity, and participation in biofilm formation of Porphyromonas gingivalis HmuY. *BMC Microbiol* (2010) 10(1):134. doi: 10.1186/1471-2180-10-134.

16. Gerritzen MJ, Martens DE, Uittenbogaard JP, Wijffels RH, Stork M. Sulfate depletion triggers overproduction of phospholipids and the release of outer membrane vesicles by Neisseria meningitidis. *Sci Rep* (2019) 9(1):1-9. doi: 10.1038/s41598-019-41233-x.

17. Elhenawy W, Bording-Jorgensen M, Valguarnera E, Haurat MF, Wine E, Feldman MF. LPS remodeling triggers formation of outer membrane vesicles in Salmonella. *mBio* (2016) 7(4):e00940-16. doi: 10.1128/mBio.00940-16.

18. Maredia R, Devineni N, Lentz P, Dallo SF, Yu J, Guentzel N, et al. Vesiculation from Pseudomonas aeruginosa under SOS. *ScientificWorldJournal* (2012) 2012. doi: 10.1100/2012/402919.

19. Lin J, Zhang W, Cheng J, Yang X, Zhu K, Wang Y, et al. A Pseudomonas T6SS effector recruits PQS-containing outer membrane vesicles for iron acquisition. *Nat Commun* (2017) 8(1):1-12. doi: 10.1038/ncomms14888.

20. Zingl FG, Kohl P, Cakar F, Leitner DR, Mitterer F, Bonnington KE, et al. Outer Membrane Vesiculation Facilitates Surface Exchange and In Vivo Adaptation of Vibrio cholerae. *Cell Host Microbe* (2020) 27(2):225-37. e8. doi: 10.1016/j.chom.2019.12.002.

21. Prados-Rosales R, Weinrick BC, Piqué DG, Jacobs WR, Casadevall A, Rodriguez GM. Role for Mycobacterium tuberculosis membrane vesicles in iron acquisition. *J Bacteriol* (2014) 196(6):1250-6. doi: 10.1128/JB.01090-13.

22. Klimentova J, Pavkova I, Horcickova L, Bavlovic J, Kofroňová O, Benada O, et al. Francisella tularensis subsp. holarctica releases differentially loaded outer membrane vesicles under various stress conditions. *Front Microbiol* (2019) 10:2304. doi: 10.3389/fmicb.2019.02304.

23. Malabirade A, Habier J, Heintz-Buschart A, May P, Godet J, Halder R, et al. The RNA complement of outer membrane vesicles from Salmonella enterica serovar typhimurium under distinct culture conditions. *Front Microbiol* (2018) 9:2015. doi: 10.3389/fmicb.2018.02015.

24. Bonnington K, Kuehn M. Protein selection and export via outer membrane vesicles. *Biochim Biophys Acta Mol Cell Res* (2014) 1843(8):1612-9. doi: 10.1016/j.bbamcr.2013.12.011.

25. Bonnington KE, Kuehn MJ. Outer membrane vesicle production facilitates LPS remodeling and outer membrane maintenance in Salmonella during environmental transitions. *mBio* (2016) 7(5):e01532-16. doi: 10.1128/mBio.01532-16.

26. Lynch JB, Schwartzman JA, Bennett BD, McAnulty SJ, Knop M, Nyholm SV, et al. Ambient pH Alters the Protein Content of Outer Membrane Vesicles, Driving Host Development in a Beneficial Symbiosis. *J Bacteriol* (2019) 201(20):e00319-19. doi: 10.1128/JB.00319-19.

27. Katsui N, Tsuchido T, Hiramatsu R, Fujikawa S, Takano M, Shibasaki I. Heat-induced blebbing and vesiculation of the outer membrane of Escherichia coli. *J Bacteriol* (1982) 151(3):1523-31. doi: 10.1128/JB.151.3.1523-1531.1982.

28. Kropinski A, Lewis V, Berry D. Effect of growth temperature on the lipids, outer membrane proteins, and lipopolysaccharides of Pseudomonas aeruginosa PAO. *J Bacteriol* (1987) 169(5):1960-6. doi: 10.1128/jb.169.5.1960-1966.1987.

29. Makin SA, Beveridge TJ. Pseudomonas aeruginosa PAO1 ceases to express serotype-specific lipopolysaccharide at 45 degrees C. *J Bacteriol* (1996) 178(11):3350-2. doi: 10.1128/jb.178.11.3350-3352.1996.

30. Murphy K, Park AJ, Hao Y, Brewer D, Lam JS, Khursigara CM. Influence of O polysaccharides on biofilm development and outer membrane vesicle biogenesis in Pseudomonas aeruginosa PAO1. *J Bacteriol* (2014) 196(7):1306-17. doi: 10.1128/JB.01463-13.

31. Walsh NP, Alba BM, Bose B, Gross CA, Sauer RT. OMP peptide signals initiate the envelope-stress response by activating DegS protease via relief of inhibition mediated by its PDZ domain. *Cell* (2003) 113(1):61-71. doi: 10.1016/s0092-8674(03)00203-4.

32. Wonderling LD, Wilkinson BJ, Bayles DO. The htrA (degP) gene of Listeria monocytogenes 10403S is essential for optimal growth under stress conditions. *Appl Environ Microbiol* (2004) 70(4):1935-43. doi: 10.1128/aem.70.4.1935-1943.2004.

33. Alba BM, Gross CA. Regulation of the Escherichia coliσE‐dependent envelope stress response. *Mol Microbiol* (2004) 52(3):613-9. doi: 10.1111/j.1365-2958.2003.03982.x.

34. Boehm M, Simson D, Escher U, Schmidt A-M, Bereswill S, Tegtmeyer N, et al. Function of serine protease HtrA in the lifecycle of the foodborne pathogen Campylobacter jejuni. *Eur J Microbiol Immunol* (2018) 8(3):70-7. doi: 10.1556/1886.2018.00011.

35. Tashiro Y, Sakai R, Toyofuku M, Sawada I, Nakajima-Kambe T, Uchiyama H, et al. Outer membrane machinery and alginate synthesis regulators control membrane vesicle production in Pseudomonas aeruginosa. *J Bacteriol* (2009) 191(24):7509-19. doi: 10.1128/JB.00722-09.

36. Baumgarten T, Sperling S, Seifert J, von Bergen M, Steiniger F, Wick LY, et al. Membrane vesicle formation as a multiple-stress response mechanism enhances Pseudomonas putida DOT-T1E cell surface hydrophobicity and biofilm formation. *Appl Environ Microbiol* (2012) 78(17):6217-24. doi: 10.1128/AEM.01525-12.

37. Gerhardt P, Smith LT, Smith GM. Sodium-driven, osmotically activated glycine betaine transport in Listeria monocytogenes membrane vesicles. *J Bacteriol* (1996) 178(21):6105-9.

38. McBroom AJ, Johnson AP, Vemulapalli S, Kuehn MJ. Outer membrane vesicle production by Escherichia coli is independent of membrane instability. *J Bacteriol* (2006) 188(15):5385-92. doi: 10.1128/JB.00498-06.

39. Mille Y, Beney L, Gervais P. Viability of Escherichia coli after combined osmotic and thermal treatment: a plasma membrane implication. *Biochim Biophys Acta Biomembr* (2002) 1567:41-8. doi: 10.1016/s0005-2736(02)00565-5.

40. Kadurugamuwa JL, Clarke AJ, Beveridge TJ. Surface action of gentamicin on Pseudomonas aeruginosa. *J Bacteriol* (1993) 175(18):5798-805. doi: 10.1128/jb.175.18.5798-5805.1993.

41. Kadurugamuwa JL, Beveridge TJ. Virulence factors are released from Pseudomonas aeruginosa in association with membrane vesicles during normal growth and exposure to gentamicin: a novel mechanism of enzyme secretion. *J Bacteriol* (1995) 177(14):3998-4008. doi: 10.1128/jb.177.14.3998-4008.1995.

42. Ciofu O, Beveridge TJ, Kadurugamuwa J, Walther-Rasmussen J, Høiby N. Chromosomal β-lactamase is packaged into membrane vesicles and secreted from Pseudomonas aeruginosa. *J Antimicrob Chemother* (2000) 45(1):9-13. doi: 10.1093/jac/45.1.9.

43. Fulsundar S, Harms K, Flaten GE, Johnsen PJ, Chopade BA, Nielsen KM. Gene transfer potential of outer membrane vesicles of Acinetobacter baylyi and effects of stress on vesiculation. *Appl Environ Microbiol* (2014) 80(11):3469-83. doi: 10.1128/AEM.04248-13.

44. Bauwens A, Kunsmann L, Karch H, Mellmann A, Bielaszewska M. Antibiotic-mediated modulations of outer membrane vesicles in enterohemorrhagic Escherichia coli O104: H4 and O157: H7. *Antimicrob Agents Chemother* (2017) 61(9):e00937-17.

45. Volgers C, Grauls GE, Hellebrand PH, Savelkoul PH, Stassen FR. Budesonide, fluticasone propionate, and azithromycin do not modulate the membrane vesicle release by THP-1 macrophages and respiratory pathogens during macrophage infection. *Inflammopharmacology* (2017) 25(6):643-51. doi: 10.1007/s10787-017-0359-7.

46. Yun SH, Park EC, Lee S-Y, Lee H, Choi C-W, Yi Y-S, et al. Antibiotic treatment modulates protein components of cytotoxic outer membrane vesicles of multidrug-resistant clinical strain, Acinetobacter baumannii DU202. *Clin Proteom* (2018) 15(1):1-11. doi: 10.1186/s12014-018-9204-2.

47. Bernier SP, Lebeaux D, DeFrancesco AS, Valomon A, Soubigou G, Coppée J-Y, et al. Starvation, together with the SOS response, mediates high biofilm-specific tolerance to the fluoroquinolone ofloxacin. *PLoS Genet* (2013) 9(1). doi: 10.1371/journal.pgen.1003144.

48. Devos S, Van Putte W, Vitse J, Van Driessche G, Stremersch S, Van Den Broek W, et al. Membrane vesicle secretion and prophage induction in multidrug‐resistant Stenotrophomonas maltophilia in response to ciprofloxacin stress. *Environ Microbiol* (2017) 19(10):3930-7. doi: 10.1111/1462-2920.13793.

49. Siebert C, Lindgren H, Ferré S, Villers C, Boisset S, Perard J, et al. Francisella tularensis: FupA mutation contributes to fluoroquinolone resistance by increasing vesicle secretion and biofilm formation. *Emerg Microbes Infect* (2019) 8(1):808-22. doi: 10.1080/22221751.2019.1615848.

50. Toyofuku M, Cárcamo-Oyarce G, Yamamoto T, Eisenstein F, Hsiao C-C, Kurosawa M, et al. Prophage-triggered membrane vesicle formation through peptidoglycan damage in Bacillus subtilis. *Nat Commun* (2017) 8(1):1-10. doi: 10.1038/s41467-017-00492-w.

51. Turnbull L, Toyofuku M, Hynen AL, Kurosawa M, Pessi G, Petty NK, et al. Explosive cell lysis as a mechanism for the biogenesis of bacterial membrane vesicles and biofilms. *Nat Commun* (2016) 7(1):1-13. doi: 10.1038/ncomms11220.

52. Rumbo C, Fernández-Moreira E, Merino M, Poza M, Mendez JA, Soares NC, et al. Horizontal transfer of the OXA-24 carbapenemase gene via outer membrane vesicles: a new mechanism of dissemination of carbapenem resistance genes in Acinetobacter baumannii. *Antimicrob Agents Chemother* (2011) 55(7):3084-90. doi: 10.1128/AAC.00929-10.

53. Jin JS, Kwon S-O, Moon DC, Gurung M, Lee JH, Kim SI, et al. Acinetobacter baumannii secretes cytotoxic outer membrane protein A via outer membrane vesicles. *PLoS One* (2011) 6(2). doi: 10.1371/journal.pone.0017027.

54. Schaar V, Paulsson M, Mörgelin M, Riesbeck K. Outer membrane vesicles shield Moraxella catarrhalis β-lactamase from neutralization by serum IgG. *J Antimicrob Chemother* (2013) 68(3):593-600. doi: 10.1093/jac/dks444.

55. Schaar V, Uddbäck I, Nordström T, Riesbeck K. Group A streptococci are protected from amoxicillin-mediated killing by vesicles containing β-lactamase derived from Haemophilus influenzae. *J Antimicrob Chemother* (2014) 69(1):117-20. doi: 10.1093/jac/dkt307.

56. Stentz R, Horn N, Cross K, Salt L, Brearley C, Livermore DM, et al. Cephalosporinases associated with outer membrane vesicles released by Bacteroides spp. protect gut pathogens and commensals against β-lactam antibiotics. *J Antimicrob Chemother* (2015) 70(3):701-9. doi: 10.1093/jac/dku466.

57. Lee J, Lee E-Y, Kim S-H, Kim D-K, Park K-S, Kim KP, et al. Staphylococcus aureus extracellular vesicles carry biologically active β-lactamase. *Antimicrob Agents Chemother* (2013) 57(6):2589-95. doi: 10.1128/AAC.00522-12.

58. Devos S, Van Oudenhove L, Stremersch S, Van Putte W, De Rycke R, Van Driessche G, et al. The effect of imipenem and diffusible signaling factors on the secretion of outer membrane vesicles and associated Ax21 proteins in Stenotrophomonas maltophilia. *Front Microbiol* (2015) 6:298. doi: 10.3389/fmicb.2015.00298.

59. Devos S, Stremersch S, Raemdonck K, Braeckmans K, Devreese B. Intra-and interspecies effects of outer membrane vesicles from Stenotrophomonas maltophilia on β-lactam resistance. *Antimicrob Agents Chemother* (2016) 60(4):2516-8. doi: 10.1128/AAC.02171-15.

60. Manning AJ, Kuehn MJ. Contribution of bacterial outer membrane vesicles to innate bacterial defense. *BMC Microbiol* (2011) 11(1):258. doi: 10.1186/1471-2180-11-258.

61. Roszkowiak J, Jajor P, Guła G, Gubernator J, Żak A, Drulis-Kawa Z, et al. Interspecies Outer Membrane Vesicles (OMVs) Modulate the Sensitivity of Pathogenic Bacteria and Pathogenic Yeasts to Cationic Peptides and Serum Complement. *Int J Mol Sci* (2019) 20(22):5577. doi: 10.3390/ijms20225577.

62. Baumgarten T, Vazquez J, Bastisch C, Veron W, Feuilloley MG, Nietzsche S, et al. Alkanols and chlorophenols cause different physiological adaptive responses on the level of cell surface properties and membrane vesicle formation in Pseudomonas putida DOT-T1E. *Appl Microbiol Biotechnol* (2012) 93(2):837-45. doi: 10.1007/s00253-011-3442-9.

63. Eberlein C, Baumgarten T, Starke S, Heipieper HJ. Immediate response mechanisms of Gram-negative solvent-tolerant bacteria to cope with environmental stress: cis-trans isomerization of unsaturated fatty acids and outer membrane vesicle secretion. *Appl Microbiol Biotechnol* (2018) 102(6):2583-93. doi: 10.1007/s00253-018-8832-9.

64. Eberlein C, Starke S, Doncel ÁE, Scarabotti F, Heipieper HJ. Quantification of outer membrane vesicles: a potential tool to compare response in Pseudomonas putida KT2440 to stress caused by alkanols. *Appl Microbiol Biotechnol* (2019) 103(10):4193-201. doi: 10.1007/s00253-019-09812-0.

65. Pinkart HC, Wolfram JW, Rogers R, White DC. Cell envelope changes in solvent-tolerant and solvent-sensitive Pseudomonas putida strains following exAposure to o-xylene. *Appl Environ Microbiol* (1996) 62(3):1129-32. doi: 10.1128/AEM.62.3.1129-1132.1996.

66. McDaniel C, Su S, Panmanee W, Lau GW, Browne T, Cox K, et al. A putative ABC transporter permease is necessary for resistance to acidified nitrite and EDTA in Pseudomonas aeruginosa under aerobic and anaerobic planktonic and biofilm conditions. *Front Microbiol* (2016) 7:291. doi: 10.3389/fmicb.2016.00291.

67. Finnegan S, Percival SL. EDTA: an antimicrobial and antibiofilm agent for use in wound care. *Adv Wound Care* (2015) 4(7):415-21. doi: EDTA: an antimicrobial and antibiofilm agent for use in wound care.

68. Matsushita K, Adachi O, Shinagawa E, Ameyama M. Isolation and characterization of outer and inner membranes from Pseudomonas aeruginosa and effect of EDTA on the membranes. *J Biochem* (1978) 83(1):171-81. doi: 10.1093/oxfordjournals.jbchem.a131888.

69. Che Y, Sanderson K, Roddam LF, Kirov SM, Reid DW. Iron-binding compounds impair Pseudomonas aeruginosa biofilm formation, especially under anaerobic conditions. *J Med Microbiol* (2009) 58(6):765-73. doi: 10.1099/jmm.0.004416-0.

70. Robertson EJ, Wolf JM, Casadevall A. EDTA inhibits biofilm formation, extracellular vesicular secretion, and shedding of the capsular polysaccharide glucuronoxylomannan by Cryptococcus neoformans. *Appl Environ Microbiol* (2012) 78(22):7977-84. doi: 10.1128/AEM.01953-12.

71. Hirayama S, Nakao R. Glycine significantly enhances bacterial membrane vesicle production: a powerful approach for isolation of LPS‐reduced membrane vesicles of probiotic Escherichia coli. *Microb Biotechnol* (2020) 13(4):1162-78. doi: 10.1111/1751-7915.13572.

72. Andreoni F, Toyofuku M, Menzi C, Kalawong R, Shambat SM, François P, et al. Antibiotics stimulate formation of vesicles in Staphylococcus aureus in both phage-dependent and-independent fashions and via different routes. *Antimicrob Agents Chemother* (2019) 63(2):e01439-18. doi: 10.1128/AAC.01439-18.

73. Crispim JS, Dias RS, Laguardia CN, Araújo LC, da Silva JD, Vidigal PMP, et al. Desulfovibrio alaskensis prophages and their possible involvement in the horizontal transfer of genes by outer membrane vesicles. *Gene* (2019) 703:50-7. doi: 10.1016/j.gene.2019.04.016.
